# Cost-effectiveness analysis of COVID-19 intervention policies using a mathematical model: an optimal control approach

**DOI:** 10.1038/s41598-023-50799-6

**Published:** 2024-01-04

**Authors:** Md Abdul Kuddus, Anip Kumar Paul, Thitiya Theparod

**Affiliations:** 1 Department of Mathematics, University of Rajshahi, Rajshahi, 6205 Bangladesh; 2 Department of Mathematics, Mahasarakham University, Maha Sarakham, 44150 Thailand

**Keywords:** Applied mathematics, Statistics

## Abstract

COVID-19 is an infectious disease that causes millions of deaths worldwide, and it is the principal leading cause of morbidity and mortality in all nations. Although the governments of developed and developing countries are enforcing their universal control strategies, more precise and cost-effective single or combination interventions are required to control COVID-19 outbreaks. Using proper optimal control strategies with appropriate cost-effectiveness analysis is important to simulate, examine, and forecast the COVID-19 transmission phase. In this study, we developed a COVID-19 mathematical model and considered two important features including direct link between vaccination and latently population, and practical healthcare cost by separation of infections into Mild and Critical cases. We derived basic reproduction numbers and performed mesh and contour plots to explore the impact of different parameters on COVID-19 dynamics. Our model fitted and calibrated with number of cases of the COVID-19 data in Bangladesh as a case study to determine the optimal combinations of interventions for particular scenarios. We evaluated the cost-effectiveness of varying single and combinations of three intervention strategies, including transmission control, treatment, and vaccination, all within the optimal control framework of the single-intervention policies; enhanced transmission control is the most cost-effective and prompt in declining the COVID-19 cases in Bangladesh. Our finding recommends that a three-intervention strategy that integrates transmission control, treatment, and vaccination is the most cost-effective compared to single and double intervention techniques and potentially reduce the overall infections. Other policies can be implemented to control COVID-19 depending on the accessibility of funds and policymakers’ judgments.

## Introduction

The SARS-CoV-2 virus responsible for COVID-19 emerged as a severe global public health crisis from the beginning of December 2019 to now and continues to pose a significant economic threat worldwide^1^. The coronavirus is recognized as a highly contagious disease that spreads so fast worldwide and all countries are grappling with the challenges of managing its severity. SARS-CoV-2 is an RNA virus that belongs to the Coronaviridae family, and the Betacoronavirus genus^2,3^. As of March 15, 2022, the total number of coronavirus-infected people worldwide is 760,897,555 with a total of 13,232,780,775 vaccine doses and the total death case record to the date is 6,874,585 though the reported figures for COVID-19 cases are likely underestimated due to asymptomatic and unreported cases^4–6^. Since 2019, people have faced several waves of attack due to emerging of different strains of the SARS-CoV-2 virus with varying levels of contagiousness and severity^7,8^.

The cause of the continuous illness is a newly discovered, highly infectious variant of severe acute respiratory syndrome coronavirus such as the Alpha variant (B.1.1.7; first identified in the UK), Beta variant (B.1.351; first identified in South Africa), Gamma variant (P.1; first identified in Brazil), Delta variant (B.1.617.2; first identified in India), Epsilon variant (B.1.427/B.1.429; first identified in California, USA), Zeta variant (P.2; first identified in Brazil), Eta variant (B.1.525; first identified in the UK and Nigeria), Theta variant (P.3; first identified in the Philippines), Iota variant (B.1.526; first identified in the US), Kappa variant (B.1.617.1; first identified in India), Lambda variant (C.37; first identified in Peru), Omicron variant (B.1.1.529; first identified in South Africa)^9,10^. To prevent the severity of coronavirus strains, all countries worldwide imposed transmission control, treatment, and vaccination strategies with multiple doses^5^. As a result, corresponding governments worldwide have taken significant measures to prevent and control the spread of the disease.

Mathematical modelling with optimal control strategies is now essential for meaningful understanding to curb the virus transmission and predict its potential outcomes. Therefore, employing appropriate optimal control strategies with proper cost-effectiveness analysis is important to simulate, examine, and forecast the COVID-19 transmission phase^11^. The vaccination strategy and enhanced treatments are the most significant parameters to curb disease transmission. Many vaccines and treatments have been authorized for emergency use globally, and research is ongoing to optimize their effectiveness^12^. In this study, we discussed the optimal control strategies and their cost-effectiveness analysis to identify more insights into the control and prevent the severity of the coronavirus.

Mathematical modelling plays a significant role in designing and predicting the transmission dynamics and identifying the parameter responsible for spreading COVID-19. The outbreak due to infectious diseases, including COVID-19, can be studied and controlled more effectively using mathematical modelling tools^13^. Several researchers are continuously trying to figure out more effective mathematical measures to control the severity and identify all sensitive parameters to mitigate and die out the novel coronavirus from the community. Several researchers proposed a mathematical model with multi-dose vaccination and stated that the proper implication of vaccination strategy is the most sensitive parameter to prevent the disease^14–18^. Ali et al.^19^ performed a global parameter sensitivity analysis using a mathematical model for COVID-19 and proposed a non-autonomous epidemic model with quarantine and isolation as time-dependent control functions. A stochastic and deterministic approach to the COVID-19 disease model was presented for forecasting the spread of COVID-19 in Africa and Europe^20^. A double-dose vaccination model is proposed to identify the disease dynamics behaviour after imposing the vaccination^21,22^. A fractional order approach is applied to the double dose vaccination model to identify significant consequences in coronavirus disease dynamics^23^. A modified SIR compartmental model for COVID-19 is presented to determine the spread of the disease with nonlinear incidence and identify the disease control policies^24^.

The COVID-19 outbreak is modelled mathematically considering the age-dependent SIR system and determined whether it is more effective to vaccinate the elderly, who are at the most risk of severe illness, or those who are more likely to spread the disease^25^. A multi-strain disease model is proposed that accounts for different disease variants with the sensitivity analysis of the model parameter^26–28^. In developing the vaccines and drugs for both prevention and treatment of the virus as well as boosting the immune responses of hosts, an animal model is discussed and evaluated for virulence of variants^29^. Parolini et al.^30^ introduce a modified version of the SUIHTER model to evaluate the spread of COVID-19 in Italy, considering the ongoing vaccination program and incorporating new variants' emergence as they become prevalent. Mengüç et al.^31^ presented a study on a mathematical model to tackle the challenges in organizing vaccination programs that arise from population heterogeneity in cities. The study aims to optimize the vaccination process by considering the available resources^31^. A multi-scale model was created that includes both population-level transmission and individual-level vaccination. The purpose was to calculate the expenses associated with hospitalization and vaccination, as well as the economic advantages of lowering COVID-19 deaths via dose-fractionation strategies in India^32^.

Optimal control strategy identifies the best intervention strategies from a non-autonomous mathematical model that can prevent virus transmission into the community while considering the available resources and economic costs. We can evaluate effective control measures which account for uncertainty and provide a robust decision-making framework. Optimal control strategies are essential for designing effective interventions that can save lives while minimizing the negative impact on society's economy. Implementing optimal control strategies can help minimize the number of infected cases, hospitalizations, and deaths caused by a coronavirus, ultimately leading to a faster and more sustained recovery from the pandemic. Kouidere et al.^33^ presented an optimal control strategy with reasonable policies for preventing COVID-19 infection by analyzing the Pontryagin's maximum principle. Cost-effectiveness analysis and three control strategies are considered, and it is found that isolating and monitoring the health of infected individuals and employing quarantine measures are the most significant to minimize the virus's spread^34^.

An optimal control strategy applied different infectious diseases including COVID-19, Dengue, and HIV, focusing on the control of disease transmission^35^. A vaccination model is proposed and analyzed for the co-spread of triple infections like the impacts of COVID-19 and dengue vaccinations on the dynamics of Zika transmission^36^. Additionally, a comprehensive optimal control and cost-effective analysis is undertaken within a co-infection model for human papillomavirus (HPV), revealing that syphilis treatment for singly infected individuals emerges as the most cost-effective approach for mitigating the burden of HPV^37^. A cost-effectiveness analysis is conducted on diabetes considering the healthy lifestyle and prevention of the development of TB by encouraging personal hygiene^38^. A novel fractional-order vaccination strategy is introduced and analyzed in the context of coronavirus and presented consequential order variations^39^.

An optimal control framework and different interventions are analyzed and presented for the mathematical modelling of drug-resistant tuberculosis in Bangladesh^40^. A deterministic Lassa fever model is analyzed with a nonlinear optimal control strategy for controlling the spread of the fever and evaluating the least costly control intervention^41,42^. Bandekar and Ghosh^43^ proposed a ten-compartment COVID-19 model with two control strategies for preventing the disease's severity in India. A non-autonomous mathematical model representing the malaria dynamics is analyzed using optimal control theory and performed the cost-effectiveness analysis for demonstrating the controlling strategy with limited resources^44–46^. A malaria transmission dynamic model with climate variation factor is discussed and delineates the optimal reduction strategies of malaria contamination with infected human treatment, bed net care, and anti-mosquito spraying inside the house^47^. A non-autonomous nonlinear deterministic model of COVID-19 is studied and performed cost-effectiveness analysis for evaluating the cost and economic health outcomes affected by the attack of the novel coronavirus^13,48–50^. They proposed four controls and analyzed fourteen optimal control strategies with numerical determination^41^. Yuan and Li^51^ performed a cost-effectiveness analysis and demonstrated a combination of three strategies to minimize the number of infected individuals.

In this article, our objective is to perform a mathematical analysis of COVID-19 and find out the most effective control measure with the cost–benefit of the health economy. Our research will provide significant insights into preventing disease outbreaks with the most cost-effective benefit. To attain our goal, we propose three effective control strategies, which are (a) transmission control strategy, (b) vaccination control strategy, (c) improving the treatment of Latent cases, and employ those strategies in the dynamic disease model. The suggested trio of approaches will significantly impact curbing the virus's transmission, leading to positive outcomes. We introduce the Pontryagin maximum principle and analyze our model to attain the optimality system. We perform numerical simulations to support the analytical determination and demonstrate several interventions for combining the proposed control strategies. Finally, the cost-effective measures are performed using the cost-effective incremental ratio (ICER) and the average cost-effectiveness ratio (ACER) to identify the most cost-effective control intervention strategy for every combination of the control strategies^52,53^.

This study is exhibited in several sections. In the Methods and materials section, we develop COVID-19 model with the mathematical formulation. The corresponding model parameters are estimated and tabulated in the parameter estimation section. The analysis and visual demonstration of the basic reproduction number and corresponding parameters are presented in the basic reproduction number section. The optimal control strategy and cost-effectiveness analysis are performed in results section. Finally, a brief discussion and conclusion are outlined at the end of this study.

## Methods and materials

### The autonomous model description

We presented a mathematical model to analyze COVID-19 spreads after imposing the vaccination strategy. Our model splits the population into seven distinct groups: the susceptible individuals (S) who can be infected through the coronavirus; those who have had first-dose vaccine (V_1_); those who have had second-dose vaccine (V_2_); the Latent compartment who are infected but not contagious (L); the virus-infected individuals who have mild disease symptoms like a low-grade fever, fatigue, muscle or body aches, loss of taste or smell, runny nose, etc., stay in the mild compartment case (M), those who have faced life-threatening complications like severe shortness of breath, persistent pain or pressure in the chest, bluish lips or face, etc. due to the virus infection stay in the critical case compartment (C), and the individuals who are recovered but previously infected, patients under treatment and isolation, dead due to the virus attack, etc., stay in the recovery compartment (R). We consider that the total population size, N(t), is constant and evenly distributed. We used this model to analyse the control of coronavirus and discuss the cost-effectiveness of varying single and combinations of several intervention strategies.

The total number of populations can be expressed as:

1 $${\text{N}}\left({\text{t}}\right)={\text{S}}\left({\text{t}}\right)+{{\text{V}}}_{1}\left({\text{t}}\right)+{{\text{V}}}_{2}\left({\text{t}}\right)+{\text{L}}\left({\text{t}}\right)+{\text{M}}\left({\text{t}}\right)+{\text{C}}\left({\text{t}}\right)+{\text{R}}\left({\text{t}}\right).$$

**Figure 1:**
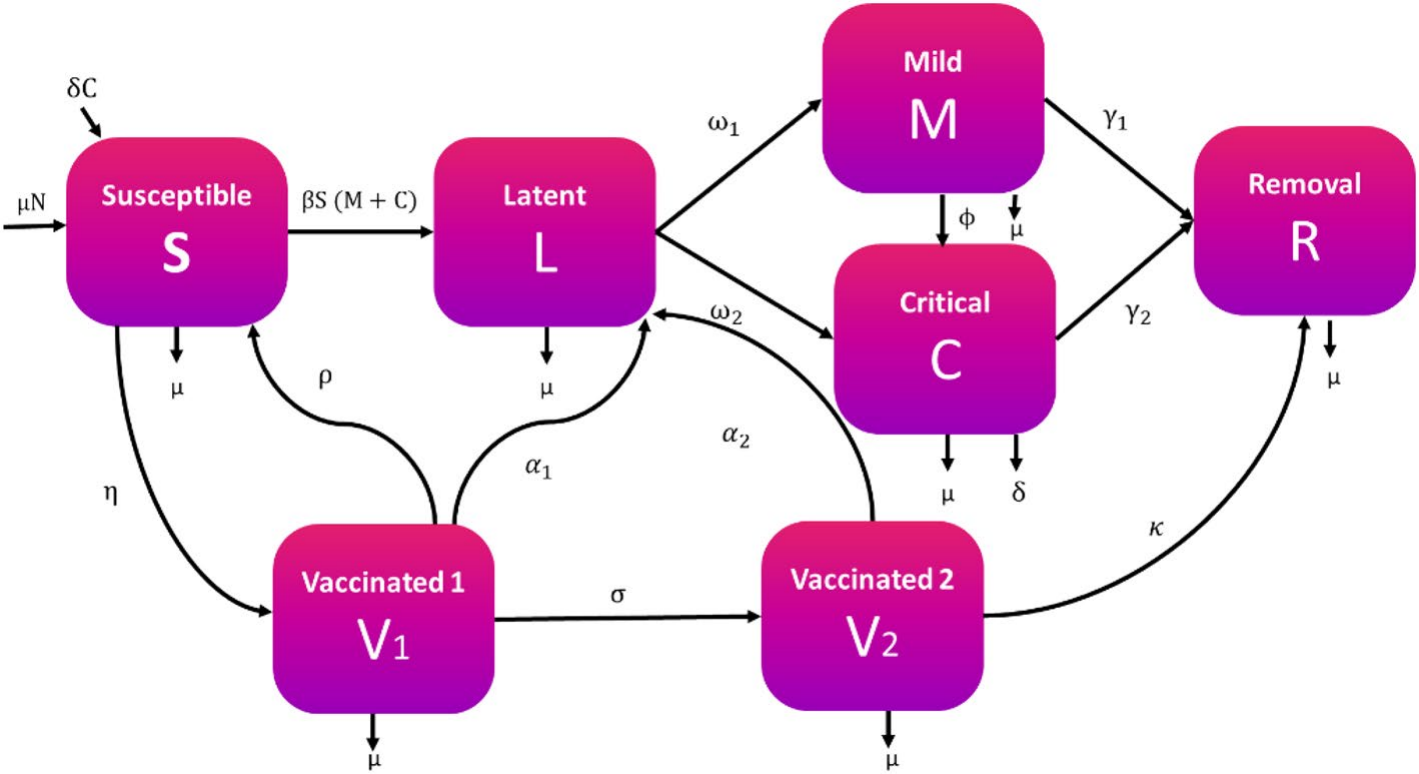
The flow diagram of the coronavirus disease dynamics.

The graphical flow diagram of the disease model is shown in the Fig. 1.

In this model, we assume that all deaths are replaced with the newborn and included into the susceptible compartment to remain the population size constant. The disease transmission rate is β. The susceptible individuals receive their first dose of vaccine with a constant rate parameter η. The first-dose vaccinated people can move to susceptible compartments with a constant rate ρ due to their immunity loss. Individuals who have been vaccinated but later lose immunity can become infected again and move to the latent class at rates ${\alpha }_{1}$ while the remaining first-dose vaccinated move to the second-dose vaccinated group V_2_ at a rate σ. There is also a possibility of being infected for the second-dose vaccinated people with no complicacy at a constant rate ${\alpha }_{2}$, whereas the rest of the individuals can move to the recovery compartment at a rate κ. Other parameters used in the model include ${\omega }_{1}$ and ${\omega }_{2}$, which represent the rates at which the latent individuals become mildly or critically infectious, respectively; the infected individuals reach the recovery stage from the mild and critical compartment with a constant rate ${\gamma }_{1}$ and ${\gamma }_{2}$, respectively; the disease co-infection rate from the mildly infected individuals to the critical individuals is ϕ; μ, which is the birth or death rate due to natural causes occurring in all states; and δ, which represents the constant per capita rate of COVID-19-related deaths.

From the above description of the disease variables, we can originate a differential equation system representing the coronavirus dynamics. The system of differential equations is:

2 $$\left.\begin{array}{c}\frac{{\text{dS}}}{{\text{dt}}}=\mu N+\rho {{\text{V}}}_{1}+\delta C-\beta S\left({\text{M}}+{\text{C}}\right)-\eta S-\mu S, \\ \frac{{{\text{dV}}}_{1}}{{\text{dt}}}=\eta S-\left(\uprho +\upsigma +{\mathrm{\alpha }}_{1}+\upmu \right){{\text{V}}}_{1}, \\ \frac{{{\text{dV}}}_{2}}{{\text{dt}}}=\sigma {{\text{V}}}_{1}-\left(\upkappa +{\mathrm{\alpha }}_{2}+\upmu \right){{\text{V}}}_{2}, \\ \frac{{\text{dL}}}{{\text{dt}}}=\beta S\left({\text{M}}+{\text{C}}\right)+{\mathrm{\alpha }}_{1}{{\text{V}}}_{1}+{\mathrm{\alpha }}_{2}{{\text{V}}}_{2}-\left({\upomega }_{1}+{\upomega }_{2}+\upmu \right)L, \\ \frac{{\text{dM}}}{{\text{dt}}}={\upomega }_{1}L-\left(\upphi +{\upgamma }_{1}+\upmu \right)M, \\ \frac{{\text{dC}}}{{\text{dt}}}={\upomega }_{2}L+\phi M-\left({\upgamma }_{2}+\updelta +\upmu \right)C, \\ \frac{{\text{dR}}}{{\text{dt}}}={\upgamma }_{1}M+{\upgamma }_{2}C+\kappa {{\text{V}}}_{2}-\mu R .\end{array}\right\}$$

The corresponding initial conditions are:

3 $${\text{S}}\left(0\right)\ge 0, {{\text{V}}}_{1}\left(0\right)\ge 0, {{\text{V}}}_{2}\left(0\right)\ge 0,\mathrm{ L}\left(0\right)\ge 0,\mathrm{ M}\left(0\right)\ge 0,\mathrm{ C}\left(0\right)\ge 0,\mathrm{ R}\left(0\right)\ge 0.$$

Positivity and boundedness of solution:

For the above system (2), we find a region of attraction which is given by Lemma 1.

#### Lemma 1

The set $D=\left\{\left(S, {V}_{1}, {V}_{2}, L, M, C, R\right)\in {\mathbb{R}}_{+}^{7}:S+{V}_{1}+{V}_{2}+L+M+C+R=N\right\}$ is invariant region of system (2).

#### Proof

Let, $$N=S+{V}_{1}+{V}_{2}+L+M+C+R$$ then

$$\frac{dN}{dt}=\frac{dS}{dt}+\frac{d{V}_{1}}{dt}+\frac{d{V}_{2}}{dt}+\frac{dL}{dt}+\frac{dM}{dt}+\frac{dC}{dt}+\frac{dR}{dt},$$

$$\frac{dN}{dt}=0,$$

Integrating

$${\text{N}}\left({\text{t}}\right)={\text{constant}}={\text{N}}.$$

This shows that the solution of system (2) toward D. Hence, D is positively invariant and solution of system (2) is bounded. The above Lemma 1 show that the solution of system (2) is non-negative and bounded.

### Basic reproduction number

The basic reproduction number (R_0_) significantly impacts monitoring the dynamics of an infectious disease outbreak and developing effective control strategies. This number quantifies a pathogen's transmission potential within a population and demonstrates the average number of secondary infections by a single infected individual in a fully susceptible population. To predict the potential impact of the coronavirus in the community and determining the necessity of the control measures, the determination of a basic reproduction number is required. The basic reproduction number depends on distinct factors, such as the pathogen's infectiousness, mode of transmission, duration of infectiousness, and population characteristics.

In this section, we demonstrate the basic reproduction number and its variation corresponding to the relative parameters value. We determined the basic reproduction number from our proposed model (2), using the next-generation matrix method. The next-generation matrix is the product of matrices $\mathcal{M}$ and ${-\mathrm{\aleph }}^{-1}$, where the matrix $\mathcal{M}$ represents the transmission components of infected states and the matrix $\mathrm{\aleph }$ describes transitions between, and out of the infected states. In this model, the infected compartments are ${\text{L}},\mathrm{M \ and \ C}$. The matrices $\mathcal{M}$ and $\mathrm{\aleph }$ for this model are given as

$$\mathcal{M}=\left(\begin{array}{ccc}0&\upbeta {{\text{S}}}^{0}&\upbeta {{\text{S}}}^{0}\\ 0& 0& 0\\ 0& 0& 0\end{array}\right),$$

and

$$\mathrm{\aleph }=\left(\begin{array}{ccc}-({\upomega }_{1}+{\upomega }_{2}+\upmu )& 0& 0\\ {\upomega }_{1}& -(\upphi +{\upgamma }_{1}+\upmu )& 0\\ {\upomega }_{2}&\upphi & -({\upgamma }_{2}+\updelta +\upmu )\end{array}\right).$$

The next-generation matrix K is given by^24,40^

$${\text{K}}=\mathcal{M}{(-\mathrm{\aleph }}^{-1})=\left(\begin{array}{ccc}0&\upbeta {{\text{S}}}^{0}&\upbeta {{\text{S}}}^{0}\\ 0& 0& 0\\ 0& 0& 0\end{array}\right)\left(\begin{array}{ccc}\frac{1}{\left({\upomega }_{1}+{\upomega }_{2}+\upmu \right)}& 0& 0\\ \frac{{\upomega }_{1}}{\left({\upomega }_{1}+{\upomega }_{2}+\upmu \right)(\upphi +{\upgamma }_{1}+\upmu )}& \frac{1}{\left(\upphi +{\upgamma }_{1}+\upmu \right)}& 0\\ \frac{{\upomega }_{1}\upphi +{\upomega }_{2}\left(\upphi +{\upgamma }_{1}+\upmu \right)}{\left({\upomega }_{1}+{\upomega }_{2}+\upmu \right)(\upphi +{\upgamma }_{1}+\upmu )({\upgamma }_{2}+\updelta +\upmu )}& \frac{\upphi }{({\upgamma }_{2}+\updelta +\upmu )\left(\upphi +{\upgamma }_{1}+\upmu \right)}& \frac{1}{({\upgamma }_{2}+\updelta +\upmu )}\end{array}\right)=\left(\begin{array}{ccc}\frac{\upbeta {{\text{S}}}^{0}\left[{\upomega }_{1}\left(\upphi +{\upgamma }_{2}+\updelta +\upmu \right)+{\upomega }_{2}\left(\upphi +{\upgamma }_{1}+\upmu \right)\right]}{\left({\upomega }_{1}+{\upomega }_{2}+\upmu \right)\left(\upphi +{\upgamma }_{1}+\upmu \right)\left({\upgamma }_{2}+\updelta +\upmu \right)}& \frac{\upbeta {{\text{S}}}^{0}\left[\left(\upphi +{\upgamma }_{2}+\updelta +\upmu \right)\right]}{\left(\upphi +{\upgamma }_{1}+\upmu \right)\left({\upgamma }_{2}+\updelta +\upmu \right)}& \frac{\upbeta {{\text{S}}}^{0}}{\left({\upgamma }_{2}+\updelta +\upmu \right)}\\ 0& 0& 0\\ 0& 0& 0\end{array}\right).$$

The spectral radius of the next generation matrix ${\text{K}}$ is considered as the basic reproduction number. Hence the mathematical expression of the basic reproduction number is:

4 $${{\text{R}}}_{0}=\frac{\upbeta {{\text{S}}}^{0}\left[{\upomega }_{1}\left(\upphi +{\upgamma }_{2}+\updelta +\upmu \right)+{\upomega }_{2}\left(\upphi +{\upgamma }_{1}+\upmu \right)\right]}{\left({\upomega }_{1}+{\upomega }_{2}+\upmu \right)\left(\upphi +{\upgamma }_{1}+\upmu \right)\left({\upgamma }_{2}+\updelta +\upmu \right)}=\frac{\mathrm{\beta \mu N}\left(\uprho +\upsigma +\upmu \right)\left[{\upomega }_{1}\left(\upphi +{\upgamma }_{2}+\updelta +\upmu \right)+{\upomega }_{2}\left(\upphi +{\upgamma }_{1}+\upmu \right)\right]}{\left(\left(\uprho +\upsigma +\upmu \right)\left(\upeta +\upmu \right)-\mathrm{\eta \rho }\right)\left({\upomega }_{1}+{\upomega }_{2}+\upmu \right)\left(\upphi +{\upgamma }_{1}+\upmu \right)\left({\upgamma }_{2}+\updelta +\upmu \right)}.$$

**Figure 2:**
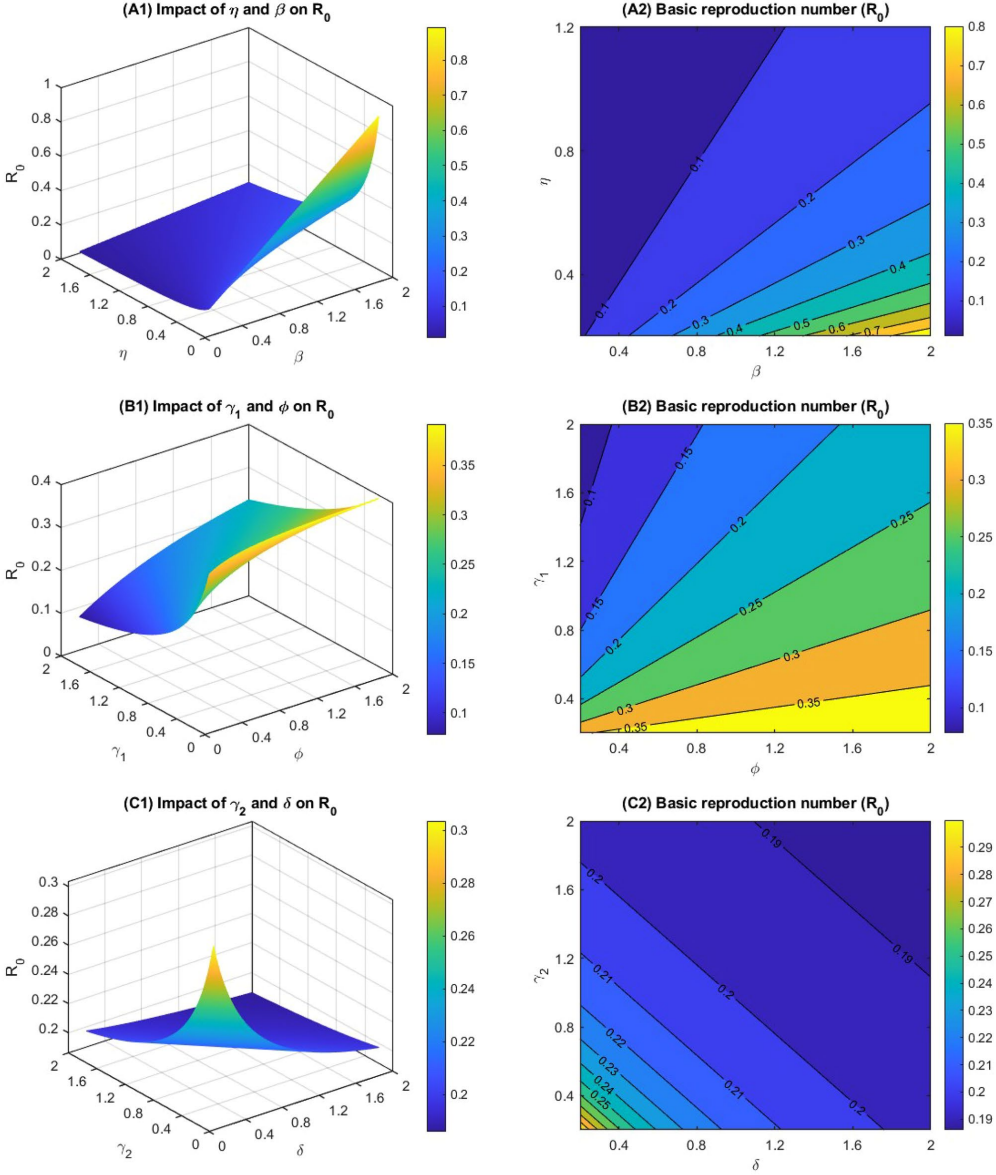

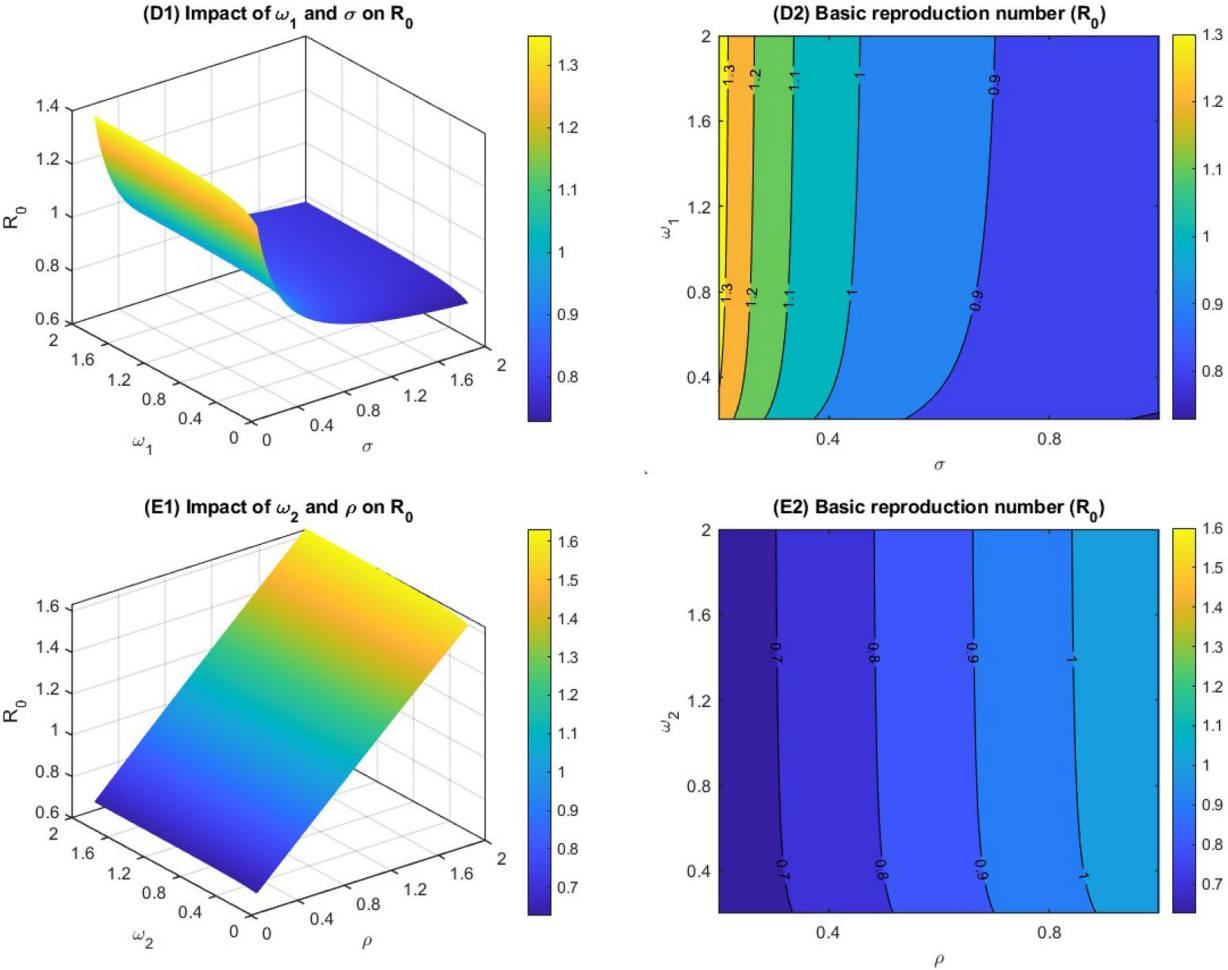
Mesh and contour plots of the basic reproduction number with the different parameters related to the ${{\text{R}}}_{0}$.

From Eq. (4), if ${R}_{0}<1$ then the disease will fade-out in the population, but if ${R}_{0}>1$ then the disease will persist in the population. To reduce ${R}_{0}$, we can vary parameters in the mathematical expression for ${R}_{0}$. ${R}_{0}$ is dependent on these parameters; $\upbeta ,\uprho ,\upsigma , {\upomega }_{1}, {\upomega }_{2},\upphi , {\upgamma }_{1}, {\upgamma }_{2},$ $\upeta ,\mathrm{ and\ \delta }$. From the Eq. (4), we can also observe that ${R}_{0}$ has positive and negative correlation with these parameters and potential repercussions of the coronavirus within the community and ascertain the imperative for controlling measures. Figure 2 represents the variation of the basic reproduction number corresponding to the impact of several model parameters which are included in Eq. (4) and depicts the result with mesh and contour plots. Our intention in performing this analysis to determine the effect of the control parameters on R_0_. In Fig. 2A1,A2, the first parameter is the number of individuals who take the first-dose vaccine (Ƞ), and the second parameter is the disease transmission rate (β). From the figure, we observe that the basic reproduction number is significantly decreasing due to the increasing first vaccination rate. This makes intuitive sense, as more individuals taking vaccination means that there are more people who are immune to the virus. However, the second parameter is disease transmission rate, which represents that a susceptible individual will become infected if they come into contact with an infected individual. From the figure, we observe that the basic reproduction number increases when the disease transmission rate increases. The higher value of disease transmission rate represents that the virus is more easily transmitted from person to person.

From Fig. 2B1,B2, we consider the recovery parameter from the mild case (γ_1_) and the co-infection rate from the mild case to the critical case (ϕ). Observing Fig. 2B1,B2, we realise that increasing the disease recovery from the mild case decreases the basic reproduction number, whereas the co-infection rate significantly increases the basic reproduction number. In Fig. 2C1,C2, we introduce a comparison of the recovery rate (γ_2_) and death rate (δ) from the critically infected case. We observe from the figure that when both the parameter's value increases, the basic reproduction number decreases, i.e., both the operator are responsible for decreasing the R_0_. In Fig. 2D1,D2, we demonstrated the combined effect of the progression rate of Latent case to mild case (ω_1_) and the second dose vaccination rate (σ). We notice from the figure that increasing the second dose vaccination rate keep a significant impact on reducing the basic reproduction number, whereas the progression rate of the Latent case to the mild case has a positive impact on increasing the R_0_.

In Fig. 2E1,E2, we introduce the effect of the progression rate from latent case to critical case (ω_2_) and the rate at which the first dose vaccinated person moves to susceptible class due to decreasing their immunity to fight against the disease (ρ). We observe that there is no significant impact of the progression rate from latent case to critical case in increasing the basic reproduction number. However, the number of individuals who lose their body immunity to fight against the coronavirus after getting the first vaccination significantly increases the R_0_. This makes intuitive sense, as more individuals joining the susceptible group means that there are more people who are not immune to the virus.

### Existence of equilibria

We found two equilibrium solutions: the disease-free equilibrium happens when ${{\text{R}}}_{0}$ is less than one i.e., ${{\text{R}}}_{0}<1$ and the disease endemic equilibrium happen when ${{\text{R}}}_{0}$ is greater than one i.e., ${{\text{R}}}_{0}>1$. We deliberate these in the following order.

Clearly, system (2) always has a disease-free equilibrium.

$$\begin{aligned} {\mathrm{E}}^{0} & = \left( {{\mathrm{S}}^{0} ,{\mathrm{~V}}_{1}^{0} ,{\mathrm{~V}}_{2}^{0} ,{\mathrm{~L}}^{0} ,{\mathrm{~M}}^{0} ,~{\mathrm{C}}^{0} } \right),\;\;{\mathrm{where,}} \\ {\mathrm{S}}^{0} & = \frac{{{{\upmu N}}\left( {{\mathrm{\rho }} + {\mathrm{\sigma }} + {\mathrm{\alpha }}_{1} + {\mathrm{\mu }}} \right)}}{{\left( {\left( {{\mathrm{\rho }} + {\mathrm{\sigma }} + {\mathrm{\alpha }}_{1} + {{\upmu }}} \right)\left( {{\mathrm{\eta }} + {\mathrm{\mu }}} \right) - {\mathrm{\eta \rho }}} \right)}}, \\ {\mathrm{V}}_{1}^{0} & = \frac{{{\mathrm{\mu \eta N}}}}{{\left( {\left( {{\mathrm{\rho }} + {\mathrm{\sigma }} + {\mathrm{\alpha }}_{1} + {\mathrm{\mu }}} \right)\left( {{\mathrm{\eta }} + {\mathrm{\mu }}} \right) - {\mathrm{\eta \rho }}} \right)}}, \\ {\mathrm{V}}_{2}^{0} & = \frac{{{\mathrm{\mu \eta \sigma N}}}}{{\left( {\left( {{\mathrm{\rho }} + {\mathrm{\sigma }} + {\mathrm{\alpha }}_{1} + {\mathrm{\mu }}} \right)\left( {{\mathrm{\eta }} + {\mathrm{\mu }}} \right) - {\mathrm{\eta \rho }}} \right)\left( {{\mathrm{\kappa }} + {\mathrm{\alpha }}_{2} + {\mathrm{\mu }}} \right)}}, \\ \end{aligned}$$

$${{\text{L}}}^{0}=0,$$

$${{\text{M}}}^{0}=0,$$

$${{\text{C}}}^{0}=0.$$

From system (2) we can also determine the endemic equilibrium

5 $$\begin{aligned} {\text{E}}^{{\text{*}}} & = \left( {{\text{S}}^{{\text{*}}} ,{\text{V}}_{1}^{{\text{*}}} ,{\text{V}}_{2}^{{\text{*}}} ,{\text{L}}^{{\text{*}}} ,{\text{M}}^{{\text{*}}} ,{\text{C}}^{{\text{*}}} } \right),\,\,{\text{where}} \\ {\text{S}}^{{\text{*}}} & = \frac{{{\text{S}}^{0} }}{{{\text{R}}_{0} }} \\ V_{1}^{*} & = \frac{{{\upeta \text{ S}}^{0} }}{{{\text{R}}_{0} \left( {{{\uprho }} + {{\upsigma }} + {{\upalpha }}_{1} + {{\upmu }}} \right)}} \\ V_{2}^{*} & = \frac{{{{\upsigma \upeta S}}^{0} }}{{{\text{R}}_{0} \left( {{{\uprho }} + {{\upsigma }} + {{\upalpha }}_{1} + {{\upmu }}} \right)\left( {{{\upkappa }} + {{\upalpha }}_{2} + {{\upmu }}} \right)}} \\ {\text{L}}^{{\text{*}}} & = \frac{{{{\upmu }\text{N}}\left( {{\text{R}}_{0} - 1} \right)\left( {\phi + {{\upgamma }}_{1} + {{\upmu }}} \right)\left( {{{\updelta }} + {{\upgamma }}_{2} + {{\upmu }}} \right)}}{{{\text{R}}_{0} \left[ {\left( {{{\upomega }}_{1} + {{\upomega }}_{2} + {{\upmu }}} \right)\left( {\phi + {{\upgamma }}_{1} + {{\upmu }}} \right)\left( {{{\updelta }} + {{\upgamma }}_{2} + {{\upmu }}} \right) - {{\updelta }}\left\{ {{{\upomega }}_{2} \left( {\phi + {{\upgamma }}_{1} + {{\upmu }}} \right) + \phi {{\upomega }}_{1} } \right\}} \right]}} \\ {\text{M}}^{{\text{*}}} & = \frac{{{{\upmu {\text{N}}\upomega }}_{1} \left( {{\text{R}}_{0} - 1} \right)\left( {{{\updelta }} + {{\upgamma }}_{2} + {{\upmu }}} \right)}}{{{\text{R}}_{0} \left[ {\left( {{{\upomega }}_{1} + {{\upomega }}_{2} + {{\upmu }}} \right)\left( {\phi + {{\upgamma }}_{1} + {{\upmu }}} \right)\left( {{{\updelta }} + {{\upgamma }}_{2} + {{\upmu }}} \right) - {{\updelta }}\left\{ {{{\upomega }}_{2} \left( {\phi + {{\upgamma }}_{1} + {{\upmu }}} \right) + \phi {{\upomega }}_{1} } \right\}} \right]}} \\ {\text{C}}^{{\text{*}}} & = \frac{{{{\upmu {\text{N}}}}\left( {{\text{R}}_{0} - 1} \right)\left( {{{\upomega }}_{2} \left( {\phi + {{\upgamma }}_{1} + {{\upmu }}} \right) + \phi {{\upomega }}_{1} } \right)}}{{{\text{R}}_{0} \left[ {\left( {{{\upomega }}_{1} + {{\upomega }}_{2} + {{\upmu }}} \right)\left( {\phi + {{\upgamma }}_{1} + {{\upmu }}} \right)\left( {{{\updelta }} + {{\upgamma }}_{2} + {{\upmu }}} \right) - {{\updelta }}\left\{ {{{\upomega }}_{2} \left( {\phi + {{\upgamma }}_{1} + {{\upmu }}} \right) + \phi {{\upomega }}_{1} } \right\}} \right]}} \\ {\text{R}}^{{\text{*}}} & = \frac{{{{\upgamma }}_{1} {{{\text{N}}\upomega }}_{1} \left( {{\text{R}}_{0} - 1} \right)\left( {{{\updelta }} + {{\upgamma }}_{2} + {{\upmu }}} \right)}}{{{\text{R}}_{0} \left[ {\left( {{{\upomega }}_{1} + {{\upomega }}_{2} + {{\upmu }}} \right)\left( {\phi + {{\upgamma }}_{1} + {{\upmu }}} \right)\left( {{{\updelta }} + {{\upgamma }}_{2} + {{\upmu }}} \right) - {{\updelta }}\left\{ {{{\upomega }}_{2} \left( {\phi + {{\upgamma }}_{1} + {{\upmu }}} \right) + \phi {{\upomega }}_{1} } \right\}} \right]}} \\ & \quad + \frac{{{{\upgamma }}_{2} {\text{N}}\left( {{\text{R}}_{0} - 1} \right)\left( {{{\upomega }}_{2} \left( {\phi + {{\upgamma }}_{1} + {{\upmu }}} \right) + \phi {{\upomega }}_{1} } \right)}}{{{\text{R}}_{0} \left[ {\left( {{{\upomega }}_{1} + {{\upomega }}_{2} + {{\upmu }}} \right)\left( {\phi + {{\upgamma }}_{1} + {{\upmu }}} \right)\left( {{{\updelta }} + {{\upgamma }}_{2} + {{\upmu }}} \right) - {{\updelta }}\left\{ {{{\upomega }}_{2} \left( {\phi + {{\upgamma }}_{1} + {{\upmu }}} \right) + \phi {{\upomega }}_{1} } \right\}} \right]}} \\ & \quad + \frac{{{{\upkappa \upsigma \upeta S}}^{0} }}{{{{\upmu {\text{R}}}}_{0} \left( {{{\uprho }} + {{\upsigma }} + {{\upalpha }}_{1} + {{\upmu }}} \right)\left( {{{\upkappa }} + {{\upalpha }}_{2} + {{\upmu }}} \right)}} \end{aligned}$$

Equation (5) displays that the endemic equilibrium ${{\text{E}}}^{*}=\left({{\text{S}}}^{*}, {{\text{V}}}_{1}^{*}, {{\text{V}}}_{2}^{*}, {{\text{L}}}^{*}, {{\text{M}}}^{*}, {{\text{C}}}^{*}, {{\text{R}}}^{*}\right)\in {\text{D}}$ (i.e., exist) if, and only if ${{\text{R}}}_{0}>1$. In the realm of infectious disease modelling, the profound impact of stability analysis on both disease-free and disease-endemic equilibrium points is paramount. This analytical framework serves as the linchpin for unravelling the ultimate trajectory of the pathogen at these equilibrium points, providing invaluable insights into the enduring dynamics of the disease within a specific population. Through these analyses, we discern that the disease-free equilibrium attains locally asymptotically stable when R_0_ is less than 1. Conversely, if R_0_ is greater than 1, i.e., ${R}_{0}>1$, the COVID-19 persists in the population. This analysis can assist us to recognise areas in the parameter space where the numerous asymptotic states are stable or unstable, thus permitting us to forecast the long-term behaviour of the COVID-19 dynamics.

### Model parameter estimation

In this section, we estimated the model parameters value introduced in the model. Parameter estimation is a significant aspect of infectious disease modelling studies, as it allows the determination of the key model parameters that help govern the spread of disease. The appropriate estimation of the model parameter is important for evaluating the effectiveness of control measures and informing public health policy. Using the most effective least square method, we analyzed real COVID-19 case data to estimate the model parameter values. The least square method is a powerful tool for parameter estimation as it can handle noisy data. It finds the line or curve that best represents the data by minimizing the distance between the actual data points and the model's predicted values. In the context of estimating parameters for the cumulative incidence of COVID-19, the following objective function is employed.

$$\mathcal{E}={\text{argmin}}\sum_{i=1}^{n}{\left({\mathcal{K}}({{\text{t}}}_{i},{\text{x}})-{{{\text{D}}}_{{\text{t}}}}_{{\text{i}}}\right)}^{2}.$$

Here, D_ti_ reveals the actual number of the infected case due to the coronavirus infection, and the number of infected individuals over time (t_i_) in the model can be reflected by the solution $\mathcal{K}$(t_i_, x), which is obtained using a set of estimated parameters (x). The number of available data points is n.

**Figure 3:**
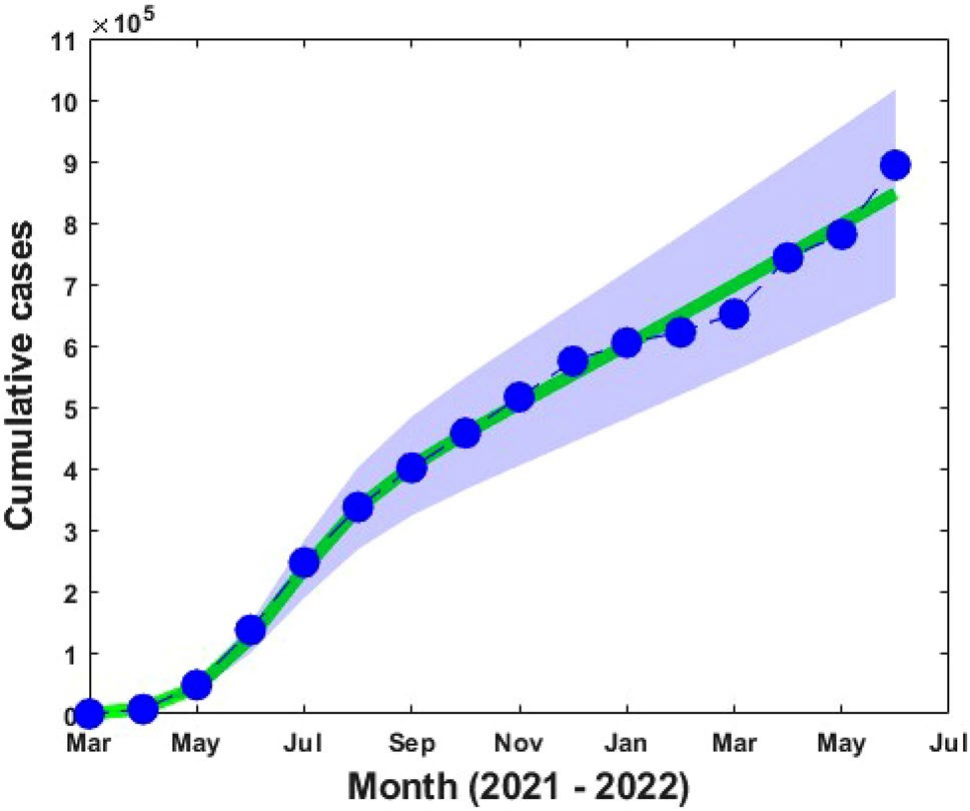
Actual reported COVID-19 incidence data (blue dots) and the model fitted curve (solid green line) with the 95% confidence interval (CI) measure in the blue-shaded limits.

In this estimation, we used the incidence data for the Bangladeshi from March 2021 to June 2022^34^. COVID-19 incidence data from Bangladesh were analyzed to understand the outbreak of different variants from March to June 2021–2022. We fitted several model parameters such as $\upbeta , {\upomega }_{1}, {\upomega }_{2},\upeta , {\mathrm{\alpha }}_{1}$ and ${\mathrm{\alpha }}_{2}$ which is shown in Table 1 using the least-squares fitting technique^54^, whereas the rest of the model parameter’s values are chosen from the well-established COVID-19 model. We considered the natural death rate (μ), which was taken as the inverse of Bangladesh's life expectancy (70 years). In incidence data and the model fitting curve with the estimated parameter values are shown in Fig. 3 with a blue dot and solid green curve, respectively, with the 95% confidence interval (CI) measured in the blue-shaded limits.

**Table 1:** The model parameter’s value.

Parameters	Description	Values	References
${\text{N}}$	Population in 2021	164,689,383	^56^
$\upmu$	Death rate	$\frac{1}{70}$	^57^
$\upbeta$	Transmission rate	$2.86\times {10}^{-6}$	Fitted
${\upomega }_{1}$	Progression rate from L to M	0.023	Fitted
${\upomega }_{2}$	Progression rate from L to C	$0.001$	Fitted
${\upgamma }_{1}$	Recovered rate of mildly infected individuals	0.02	^58^
${\upgamma }_{2}$	Recovered rate of critically infected individuals	0.01	^58^
$\upphi$	Transfer rate from mild to critical compartment	0.3	^58^
$\uprho$	The rate at which first dose vaccinated person move to susceptible class	0.2	^59^
$\updelta$	Death rate of critically infected individuals	0.125	^60^
$\upeta$	First dose vaccination rate	1.02	Fitted
${\alpha }_{1}$	Loss of immunity from first dose vaccinated person	0.0053	Fitted
${\alpha }_{2}$	Loss of immunity from second dose vaccinated person	0.0204	Fitted
$\upsigma$	Second dose vaccination rate	0.90	Assumed
$\upkappa$	Recovered rate from second dose vaccinated individuals	0.80	^60^

### Ethical approval

This study is based on aggregated measles surveillance data in Bangladesh provided by the Directorate General of Health Services (DGHS). No confidential information was included because mathematical analyses were performed at the aggregate level. We complied data from the publicly available website https://dashboard.dghs.gov.bd/pages/covid19.php.

## Results

### Optimal control strategy

In this section, we performed an optimal control strategy and implemented three time-dependent control variables to explore their effectiveness and cost-effective analysis in controlling the spread of COVID-19 in Bangladesh. From the analysis of the basic reproduction number with corresponding rate parameters value, we identified several sensitive parameters to increase R_0_. That is why we propose three time-dependent control variables ${{\text{u}}}_{1}\left({\text{t}}\right), {{\text{u}}}_{2}({\text{t}})$ and ${{\text{u}}}_{3}({\text{t}})$ to control disease transmission into the community, and the controls are defined as follows:

i) ${{\text{u}}}_{1}({\text{t}})$ designates the transmission control strategy that is the exertion at preventing the coronavirus transmission from Susceptible to Latent, Mild, and Critical cases population. This can be contacted through public health encouragement for social distancing, mask-wearing, good personal hygiene, reducing participation in outdoor activities, diagnosis campaigns, and education programs for public health. Noting that ${{\text{u}}}_{1}\left({\text{t}}\right)=1$ designates the strategy successfully protects against COVID-19 infection while ${{\text{u}}}_{1}\left({\text{t}}\right)=0$ represents strategy failure.
ii) ${{\text{u}}}_{2}({\text{t}})$ represents the vaccination control strategy, it is presumed that the number of vaccines available during this time period and they are all administrated and used completely. If ${{\text{u}}}_{2}\left({\text{t}}\right)=1$, then the control strategy is efficiently used while ${{\text{u}}}_{2}\left({\text{t}}\right)=0$ means the lack of a control strategy.
iii) ${{\text{u}}}_{3}({\text{t}})$ designates control variables to improve the treatment of the Mild and Critical cases with a view to confirm the quick provision of extra treatment, including providing comfort measures to release COVID-19 symptoms and preventing complications. Observing ${{\text{u}}}_{3}\left({\text{t}}\right)=1$, then the control strategy is efficiently treating the COVID-19-infected population while ${{\text{u}}}_{3}\left({\text{t}}\right)=0$ means the strategy failure.

Consequently, the optimal control model with the three above-mentioned time-dependent variables is given by the following non-linear differential equations:

6 $$\left.\begin{array}{c}\frac{{\text{dS}}}{{\text{dt}}}=\mu N+\rho {{\text{V}}}_{1}+\delta C-\left(1-{{\text{u}}}_{1}\left({\text{t}}\right)\right)\beta S\left({\text{M}}+{\text{C}}\right)-\eta S-\mu S, \\ \frac{{{\text{dV}}}_{1}}{{\text{dt}}}=\eta S-\left(\uprho +{\mathrm{\alpha }}_{1}+\upmu \right){{\text{V}}}_{1}-\sigma \left(1+{{\text{u}}}_{2}\left({\text{t}}\right)\right){{\text{V}}}_{1}, \\ \frac{{{\text{dV}}}_{2}}{{\text{dt}}}=\sigma \left(1+{{\text{u}}}_{2}\left({\text{t}}\right)\right){{\text{V}}}_{1}-\kappa \left(1+{{\text{u}}}_{2}\left({\text{t}}\right)\right){{\text{V}}}_{2}-\left({\mathrm{\alpha }}_{2}+\upmu \right){{\text{V}}}_{2}, \\ \frac{{\text{dL}}}{{\text{dt}}}=\left(1-{{\text{u}}}_{1}\left({\text{t}}\right)\right)\beta S\left({\text{M}}+{\text{C}}\right)+\left({\mathrm{\alpha }}_{1}+{\mathrm{\alpha }}_{2}\right)L-\left({\upomega }_{1}+{\upomega }_{2}+\upmu \right)L, \\ \frac{{\text{dM}}}{{\text{dt}}}={\upomega }_{1}L-\left(\upphi +\upmu \right)M-\left(1+{{\text{u}}}_{3}\left({\text{t}}\right)\right){\upgamma }_{1}M, \\ \frac{{\text{dC}}}{{\text{dt}}}={\upomega }_{2}L+\phi M-\left(\updelta +\upmu \right)M-{\upgamma }_{2}\left(1+{{\text{u}}}_{3}\left({\text{t}}\right)\right)C, \\ \frac{{\text{dR}}}{{\text{dt}}}={\upgamma }_{1}\left(1+{{\text{u}}}_{3}\left({\text{t}}\right)\right)M+{\upgamma }_{2}\left(1+{{\text{u}}}_{3}\left({\text{t}}\right)\right)C+\kappa \left(1+{{\text{u}}}_{2}\left({\text{t}}\right)\right){{\text{V}}}_{2}-\mu R.\end{array}\right\}$$

The objective of giving the three control variables is to pursue the optimal solution essential to minimize the numbers of Latent, Mild, and Critical cases at minimum cost. Hence, the objective function for this optimal control problem is given by

7 $$\mathrm{J }\left({{\text{u}}}_{1}^{*}, {{\text{u}}}_{2}^{*}, {{\text{u}}}_{3}^{*}\right)=\underset{0\le {{\text{u}}}_{1}, {{\text{u}}}_{2}, {{\text{u}}}_{3}\le 1}{{\text{min}}}{\int }_{{{\text{T}}}_{0}}^{{{\text{T}}}_{{\text{f}}}}\left({{\text{P}}}_{1}{\text{L}}+{{\text{P}}}_{2}{\text{M}}+{{\text{P}}}_{3}{\text{C}}+\frac{1}{2}\left({{\text{Q}}}_{1}{{\text{u}}}_{1}^{2}\left({\text{t}}\right)+{{\text{Q}}}_{2}{{\text{u}}}_{2}^{2}\left({\text{t}}\right)+{{\text{Q}}}_{3}{{\text{u}}}_{3}^{2}({\text{t}})\right)\right){\text{dt}}.$$

where, constants ${{\text{P}}}_{{\text{i}}},\mathrm{ i}=1, 2, 3$ are positive weights crucial to balance the objective function. Following other studies on optimal control problems^25,30–32^, quadratic cost on the controls are selected to guarantee the control has only one extremum (i.e., maximum or minimum), where $\frac{1}{2}{{\text{Q}}}_{1}{{\text{u}}}_{1}^{2}({\text{t}})$ is the total cost of implementing the transmission, and $\frac{1}{2}{{\text{Q}}}_{2}{{\text{u}}}_{2}^{2}({\text{t}})$ is the total cost of vaccination and $\frac{1}{2}{{\text{Q}}}_{3}{{\text{u}}}_{3}^{2}({\text{t}})$ is the total cost of treatment for Latent, Mild, and Critical populations over the time interval $\left[{{\text{T}}}_{0}, {{\text{T}}}_{{\text{f}}}\right]$ (where the initial time ${{\text{T}}}_{0}=0$, final time ${{\text{T}}}_{{\text{f}}}=30$ months period).

Precisely, the optimal control strategy ${{\text{u}}}^{*}=\left({{\text{u}}}_{1}^{*}, {{\text{u}}}_{2}^{*}, {{\text{u}}}_{3}^{*}\right)$ is required such that

8 $${\text{J}}\left({{\text{u}}}_{1}^{*}, {{\text{u}}}_{2}^{*}, {{\text{u}}}_{3}^{*}\right)={\text{min}}\left\{{\text{J}}\left({{\text{u}}}_{1}, {{\text{u}}}_{2}, {u}_{3}\right):{{\text{u}}}_{1}, {{\text{u}}}_{2,}{{\text{u}}}_{3}\in {\text{U}}\right\}.$$

where ${\text{U}}$ is the non-empty control set defined by

$${\text{U}}=\left\{\left({{\text{u}}}_{1}, {{\text{u}}}_{2},{{\text{u}}}_{3}\right):\left({{\text{u}}}_{1}\left({\text{t}}\right), {{\text{u}}}_{2}\left({\text{t}}\right),{{\text{u}}}_{3}\left({\text{t}}\right)\right)\mathrm{ are\ measurable\ with\ }0\le {{\text{u}}}_{1}, {{\text{u}}}_{2}, {{\text{u}}}_{3}\le 1\mathrm\ { for\ t}\in [{{\text{T}}}_{0},{{\text{T}}}_{{\text{f}}}]\right\}$$

Therefore, to adjust the necessary conditions that the optimal control strategy $\left({{\text{u}}}_{1}^{*}, {{\text{u}}}_{2}^{*}, {{\text{u}}}_{3}^{*}\right)$ must satisfy, Pontryagin’s maximum principle^61^, which changes into the optimal control problem (8) subject to the model (6) that minimizes pointwise a Hamiltonian ${{\text{H}}}$, with respect to the control measures. This Hamiltonian is given as

9 $$\begin{aligned} {\text{H}} & = {\text{P}}_{1} {\text{L}} + {\text{P}}_{2} {\text{M}} + {\text{P}}_{3} {\text{C}} + \frac{1}{2}\left( {{\text{Q}}_{1} {\text{u}}_{1}^{2} \left( {\text{t}} \right) + {\text{Q}}_{2} {\text{u}}_{2}^{2} \left( {\text{t}} \right) + {\text{Q}}_{3} {\text{u}}_{3}^{2} \left( {\text{t}} \right)} \right) \\ & + \uplambda _{{\text{S}}} \left( {\upmu {\text{N}} + \uprho {\text{V}}_{1} + \updelta {\text{C}} - \left( {1 - {\text{u}}_{1} \left( {\text{t}} \right)} \right)\upbeta {\text{S}}\left( {{\text{M}} + {\text{C}}} \right) - \upeta {\text{S}} - \upmu {\text{S}}~~~} \right) \\ & + \uplambda _{{{\text{V}}_{1} }} \left( {\eta {\text{S}} - \left( {\uprho + \upalpha _{1} + \upmu } \right){\text{V}}_{1} - \upsigma ~\left( {1 + u_{2} \left( t \right)} \right){\text{V}}_{1} } \right) \\ & + \uplambda _{{{\text{V}}_{2} }} \left( {\upsigma \left( {1 + {\text{u}}_{2} \left( {\text{t}} \right)} \right)V_{1} - \upkappa \left( {1 + {\text{u}}_{2} \left( t \right)} \right)V_{2} - \left( {\upalpha _{2} + \upmu } \right)} \right)V_{2} \\ & + \uplambda _{{\text{L}}} \left( {\left( {1 - u_{1} \left( {\text{t}} \right)} \right)\upbeta S\left( {M + C} \right) + \left( {\upalpha _{1} + \upalpha _{2} } \right)L - \left( {\upomega _{1} + \upomega _{2} + \upmu } \right)L} \right) \\ & + \uplambda _{{\text{M}}} \left( {\upomega _{1} {\text{L}} - \left( {\phi + \upmu } \right){\text{M}} - \left( {1 + {\text{u}}_{3} \left( {\text{t}} \right)} \right)\upgamma _{1} {\text{M}}} \right) \\ & + \uplambda _{{\text{C}}} \left( {\upomega _{2} {\text{L}} + \phi {\text{M}} - \left( {\updelta + \upmu } \right)M - \upgamma _{2} \left( {1 + u_{3} \left( t \right)} \right)C} \right) \\ & + \uplambda _{{\text{R}}} \left( {\upgamma _{1} \left( {1 + {\text{u}}_{3} \left( {\text{t}} \right)} \right){\text{M}} + \upgamma _{2} \left( {1 + {\text{u}}_{3} \left( {\text{t}} \right)} \right){\text{C}} + \upkappa \left( {1 + {\text{u}}_{2} \left( {\text{t}} \right)} \right){\text{V}}_{2} - \upmu {\text{R}}} \right). \end{aligned}$$

where, ${\uplambda }_{{\text{i}}},\mathrm{ i}={\text{S}}, {{\text{V}}}_{1}, {{\text{V}}}_{2}, {\text{L}},\mathrm{ M},\mathrm{ C},\mathrm{ R},$ represent the adjoint variables allied with the state variables of the model (6). The predictable consequence for minimizing the control problem as implemented in^32,44^ is adjusted below. Now using Pontryagin’s maximum principle, we acquire the following theorem.

#### Theorem

Given that $\left({{\text{u}}}_{1}^{*}, {{\text{u}}}_{2}^{*}, {{\text{u}}}_{3}^{*}\right)$ minimizes the objective function (7) subject to the corresponding system (6), then the adjoint variables ${\text{S}}, {{\text{V}}}_{1}, {{\text{V}}}_{2},\mathrm{ L},\mathrm{ M},\mathrm{ C},\mathrm{ R},$ satisfy the following system.

10 $$\left.\begin{array}{c}\frac{{\text{d}}{\uplambda }_{{\text{S}}}}{{\text{dt}}}={\uplambda }_{{\text{S}}}\left(\left(1-{{\text{u}}}_{1}\right)\upbeta \left({\text{M}}+{\text{C}}\right)+\left(\upeta +\upmu \right)\right)-{\uplambda }_{{{\text{V}}}_{1}}\eta -{\uplambda }_{{\text{L}}}\left(1-{{\text{u}}}_{1}\right)\beta \left({\text{M}}+{\text{C}}\right), \\ \frac{{\text{d}}{\uplambda }_{{{\text{V}}}_{1}}}{{\text{dt}}}=-{\uplambda }_{{\text{S}}}\rho +{\uplambda }_{{{\text{V}}}_{1}}\left(\left(\uprho +{\mathrm{\alpha }}_{1}+\upmu \right)+\upsigma \left(1+{{\text{u}}}_{2}\right)\right)-{\uplambda }_{{{\text{V}}}_{2}}\sigma \left(1+{{\text{u}}}_{2}\right), \\ \frac{{\text{d}}{\uplambda }_{{{\text{V}}}_{2}}}{{\text{dt}}}={\uplambda }_{{{\text{V}}}_{2}}\left(\upkappa \left(1+{{\text{u}}}_{2}\right)+\left({\mathrm{\alpha }}_{2}+\upmu \right)\right)-{\uplambda }_{{\text{R}}}\kappa \left(1+{{\text{u}}}_{2}\right), \\ \frac{{\text{d}}{\uplambda }_{{\text{L}}}}{{\text{dt}}}=-{{\text{P}}}_{1}+{\uplambda }_{{\text{L}}}\left(\left({\upomega }_{1}+{\upomega }_{2}+\upmu \right)-\left({\mathrm{\alpha }}_{1}+{\mathrm{\alpha }}_{2}\right)\right)-{\uplambda }_{{\text{M}}}{\upomega }_{1}-{\uplambda }_{{\text{C}}}{\upomega }_{2}, \\ \frac{{\text{d}}{\uplambda }_{{\text{M}}}}{{\text{dt}}}=-{{\text{P}}}_{2}+{\uplambda }_{{\text{S}}}\left(1-{{\text{u}}}_{1}\right)\beta S-{\uplambda }_{{\text{L}}}\left(1-{{\text{u}}}_{1}\right)\beta S+{\uplambda }_{{\text{M}}}\left(\left(\upphi +\upmu \right)+{\upgamma }_{1}\left(1+{{\text{u}}}_{3}\right)\right)-{\uplambda }_{{\text{C}}}\left(\upphi -\left(\updelta +\upmu \right)\right)-{\uplambda }_{{\text{R}}}{\upgamma }_{1}\left(1+{{\text{u}}}_{3}\right),\\ \frac{{\mathrm{d\lambda }}_{{\text{C}}}}{{\text{dt}}}= -{{\text{P}}}_{3}+{\uplambda }_{{\text{S}}}\left(1-{{\text{u}}}_{1}\right)\beta S-{\uplambda }_{{\text{L}}}\left(1-{{\text{u}}}_{1}\right)\beta S+{\uplambda }_{{\text{C}}}{\upgamma }_{2}\left(1+{{\text{u}}}_{3}\right)-{\uplambda }_{{\text{R}}}{\upgamma }_{2}\left(1+{{\text{u}}}_{3}\right), \\ \frac{{\text{d}}{\uplambda }_{{\text{R}}}}{{\text{dt}}}=\mu {\uplambda }_{{\text{R}}}.\end{array}\right\}$$

with the terminal (transversality) conditions

11 $${\uplambda }_{{\text{i}}}\left({{\text{T}}}_{{\text{f}}}\right)=0,\mathrm{ i}={\text{S}}, {{\text{V}}}_{1}, {{\text{V}}}_{2},\mathrm{ L},\mathrm{ M},\mathrm{ C},\mathrm{ R}.$$

Further, the optimal control pair $({{\text{u}}}_{1}^{*}, {{\text{u}}}_{2}^{*}, {{\text{u}}}_{3}^{*})$ is given as follows.

12 $$\begin{gathered} {\text{u}}_{1}^{*} = {\text{max}}\left\{ {0,{\text{ min}}\left\{ {1,\frac{{{\beta S}\left( {{\text{M}} + {\text{C}}} \right)\left( {{\uplambda }_{{\text{L}}} - {\uplambda }_{{\text{S}}} } \right)}}{{{\text{Q}}_{1} }}} \right\}} \right\}, \hfill \\ {\text{u}}_{2}^{*} = {\text{max}}\left\{ {0,{\text{ min}}\left\{ {1,\frac{{\left( {\lambda_{{V_{1} }} - \lambda_{{V_{2} }} } \right)\sigma V_{1} + \left( {\lambda_{{V_{2} }} - \lambda_{R} } \right)\kappa V_{2} }}{{Q_{2} }}} \right\}} \right\}, \hfill \\ {\text{u}}_{3}^{*} = {\text{max}}\left\{ {0,{\text{ min}}\left\{ {1,\frac{{\left( {\lambda_{M} - \lambda_{R} } \right)\gamma_{1} M + \left( {\lambda_{C} - \lambda_{R} } \right)\gamma_{2} C}}{{{\text{Q}}_{3} }}} \right\}} \right\}.{ } \hfill \\ \end{gathered}$$

**Proof** The existence of the optimal controls ${{\text{u}}}_{1 }^{*}, {{\text{u}}}_{2}^{*}$ and ${{\text{u}}}_{3}^{*}$ such that

${\text{J}}\left({{\text{u}}}_{1}^{*}\left({\text{t}}\right), {{\text{u}}}_{2}^{*}\left(t\right), {{\text{u}}}_{3}^{*}(t)\right)={}_{{\text{U}}}{}^{{\text{min}}}\mathrm{ J}({{\text{u}}}_{1}, {{\text{u}}}_{2}, {{\text{u}}}_{3})$ with state system (6) is given by the convexity of the objective function integrand. The adjoint equations and transversality conditions are achieved by Pontryagin’s Maximum Principle^61^. Differentiation of Hamiltonian ${\text{H}}$ for the state variables gives the following system,

$$\frac{{\text{d}}{\uplambda }_{{\text{S}}}}{{\text{dt}}}=-\frac{\partial {\text{H}}}{\partial {\text{S}}},$$

$$\frac{{\text{d}}{\uplambda }_{{{\text{V}}}_{1}}}{{\text{dt}}}=-\frac{\partial {\text{H}}}{\partial {{\text{V}}}_{1}},$$

$$\frac{{\text{d}}{\uplambda }_{{V}_{2}}}{{\text{dt}}}=-\frac{\partial {\text{H}}}{\partial {{\text{V}}}_{2}},$$

$$\frac{{\text{d}}{\uplambda }_{L}}{{\text{dt}}}=-\frac{\partial {\text{H}}}{\partial {\text{L}}},$$

$$\frac{{\text{d}}{\uplambda }_{M}}{{\text{dt}}}=-\frac{\partial {\text{H}}}{\partial {\text{M}}},$$

$$\frac{{\text{d}}{\uplambda }_{{\text{C}}}}{{\text{dt}}}=-\frac{\partial {\text{H}}}{\partial {\text{C}}},$$

$$\frac{{\text{d}}{\uplambda }_{R}}{{\text{dt}}}=-\frac{\partial {\text{H}}}{\partial {\text{R}}},$$

with $${\uplambda }_{{\text{i}}}=0$$, for $${\text{i}}={\text{S}}, {{\text{V}}}_{1}, {{\text{V}}}_{2},\mathrm{ L},\mathrm{ M},\mathrm{ C},\mathrm{ R}$$.

Optimal controls ${{\text{u}}}_{1}^{*}\left({\text{t}}\right), {{\text{u}}}_{2}^{*}\left({\text{t}}\right)$ and ${{\text{u}}}_{3}^{*}\left({\text{t}}\right)$ are derived by the following optimality conditions,

$$\frac{\partial {\text{H}}}{\partial {{\text{u}}}_{1}}={Q}_{1}{u}_{1}^{*}+{\uplambda }_{{\text{S}}}\mathrm{\beta S}\left({\text{M}}+{\text{C}}\right)-{\uplambda }_{{\text{L}}}\mathrm{\beta S}({\text{M}}+{\text{C}})=0,$$

$$\frac{\partial {\text{H}}}{\partial {{\text{u}}}_{2}}={{\text{Q}}}_{2}{{\text{u}}}_{2}^{*}-{{\uplambda }_{{\text{V}}}}_{1}\upsigma {{\text{V}}}_{1}-{{\uplambda }_{{\text{V}}}}_{2}\upkappa {{\text{V}}}_{2}+{\uplambda }_{{\text{R}}}\upkappa {{\text{V}}}_{2}+{{\uplambda }_{{\text{V}}}}_{2}\upsigma {{\text{V}}}_{1}=0,$$

$$\frac{\partial {\text{H}}}{\partial {{\text{u}}}_{3}}={{\text{P}}}_{3}{{\text{u}}}_{3}^{*}-{\uplambda }_{{\text{M}}}{\upgamma }_{1}{\text{M}}-{\uplambda }_{{\text{C}}}{\upgamma }_{2}{\text{C}}+{\uplambda }_{{\text{R}}}{\upgamma }_{1}{\text{M}}+{\uplambda }_{{\text{R}}}{\upgamma }_{2}{\text{C}}=0,$$

at ${{\text{u}}}_{1}^{*}\left({\text{t}}\right), {{\text{u}}}_{2}^{*}\left({\text{t}}\right)$ and ${{\text{u}}}_{3}^{*}\left({\text{t}}\right)$ on the set ${\text{U}}$. On this set

$${{\text{u}}}_{1}^{*}\left({\text{t}}\right)=\frac{\mathrm{\beta S}({\text{M}}+{\text{C}})({\uplambda }_{{\text{L}}}-{\uplambda }_{{\text{S}}})}{{{\text{Q}}}_{1}},$$

$${{\text{u}}}_{2}^{*}\left({\text{t}}\right)=\frac{\left({\uplambda }_{{{\text{V}}}_{1}}-{\uplambda }_{{{\text{V}}}_{2}}\right)\upsigma {{\text{V}}}_{1}+\left({\uplambda }_{{{\text{V}}}_{2}}-{\uplambda }_{{\text{R}}}\right)\upkappa {{\text{V}}}_{2}}{{{\text{Q}}}_{2}},$$

$${{\text{u}}}_{3}^{*}\left({\text{t}}\right)=\frac{\left({\uplambda }_{{\text{M}}}-{\uplambda }_{{\text{R}}}\right){\upgamma }_{1}{\text{M}}+\left({\uplambda }_{{\text{C}}}-{\uplambda }_{{\text{R}}}\right){\upgamma }_{2}{\text{C}}}{{{\text{Q}}}_{3}}.$$

This ends the proof.

Here, we employed the Runge–Kutta fourth-order forward and backward technique using MATLAB programming language to solve the consequent optimality system, which contains (6) and (10) with the characterization (12) within the period of [0, 30] months. The weight constants implemented for corresponding to the objective function (7) are designated to confirm that no term dictates the other. Therefore, we used identical weight constants to minimize Latent, Mild, and Critical classes so that ${{\text{P}}}_{1}={{\text{P}}}_{2}={{\text{P}}}_{3}=1.$ Under other circumstances, the weight constants for decisive efforts or cost crucial to implement the controls are relatively different, and outcomes in values for ${{\text{Q}}}_{1}=100, {{\text{Q}}}_{2}=1000$ and ${{\text{Q}}}_{3}=1050$ are consistent with previous modeling studies^62^. Details of the numerical technique for simulating the achieved optimality system are contained^63^.

**Figure 4:**
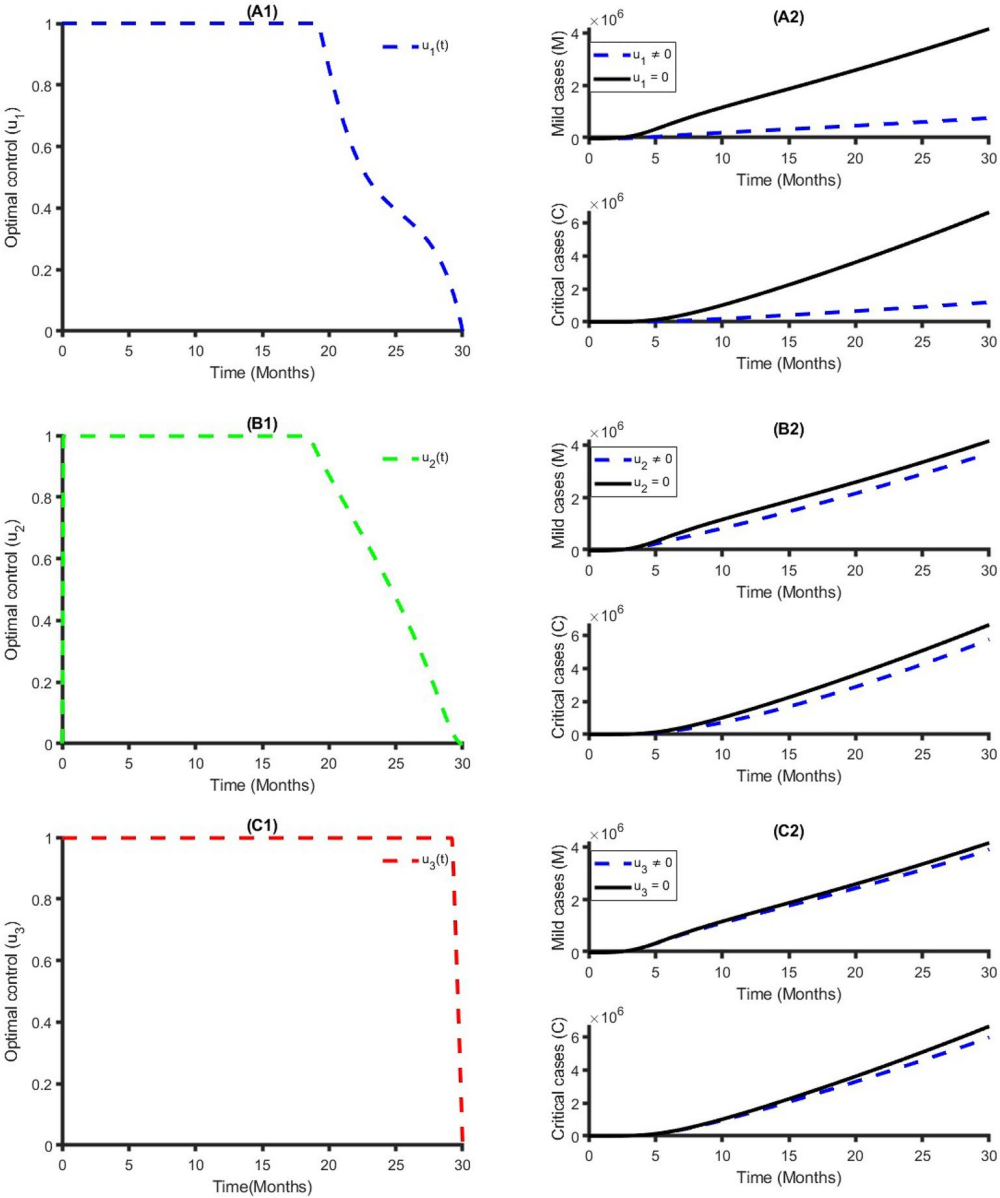
Single intervention strategy and its impacts on the COVID-19 cases in Bangladesh.

Figure 4 establishes how transmission control $({{\text{u}}}_{1})$, vaccination $({{\text{u}}}_{2})$ and treatment $({{\text{u}}}_{3})$ control strategies that affect the spread of COVID-19 in Bangladesh. As shown in Fig. 4, to minimize the objective function (7), the optimal control ${{\text{u}}}_{1}({\text{t}})$, ${{\text{u}}}_{2}({\text{t}})$ and ${{\text{u}}}_{3}({\text{t}})$ are continued at the maximum level (i.e., 100%) for about 18 months, 17 months, and 30 months respectively, for the Bangladesh population before relaxing to the minimum in the final time. Also, as expected, the number of COVID-19 infectious individuals is reduced when control is in place. We observed that the treatment control strategy has a small impact on Mild and Critical cases, while the transmission control strategy has a high impact on reducing the burden of COVID-19 cases in Bangladesh.

**Figure 5:**
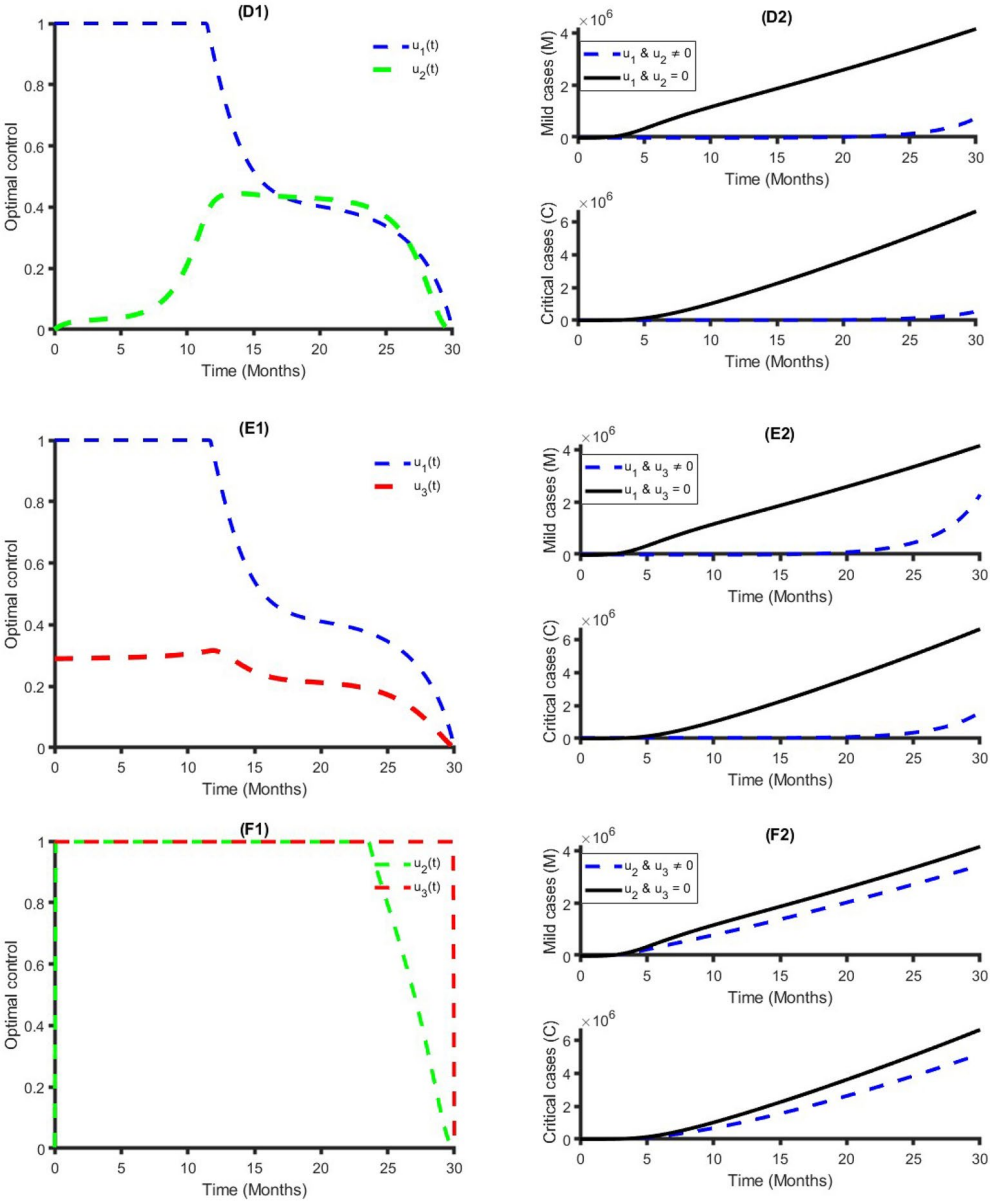
Double intervention strategy and its impacts on the COVID-19 cases in Bangladesh.

Figure 5 represents the implication of double intervention strategies, including transmission control and vaccination, transmission control and treatment, and vaccination and treatment. Each of the interventions resulted in decreasing the number of COVID-19 cases during the time period. The analysis shows that a combination of transmission control and vaccination is the best dual intervention strategy for reducing the number of the total number of COVID-19 cases and minimum cost compared to other dual intervention strategies (see Table 2 and Fig. 5). An alternative, a combination of transmission control and treatment rate, is another option.

**Table 2:** ICER and ACER in the order of COVID-19 cases averted by single control measures.

Control measures	Total infected averted	Total cost	ICER	ACER
${{\text{S}}}_{1}$	$7.3961\times {10}^{8}$	$1.2910\times {10}^{6}$	$0.0017$	$0.0017$
${{\text{S}}}_{2}$	$9.0816\times {10}^{7}$	$9.5607\times {10}^{8}$	$-1.47$	$10.5276$
${{\text{S}}}_{3}$	$5.4928\times {10}^{6}$	$1.1152\times {10}^{9}$	$-1.87$	$203$

**Figure 6:**
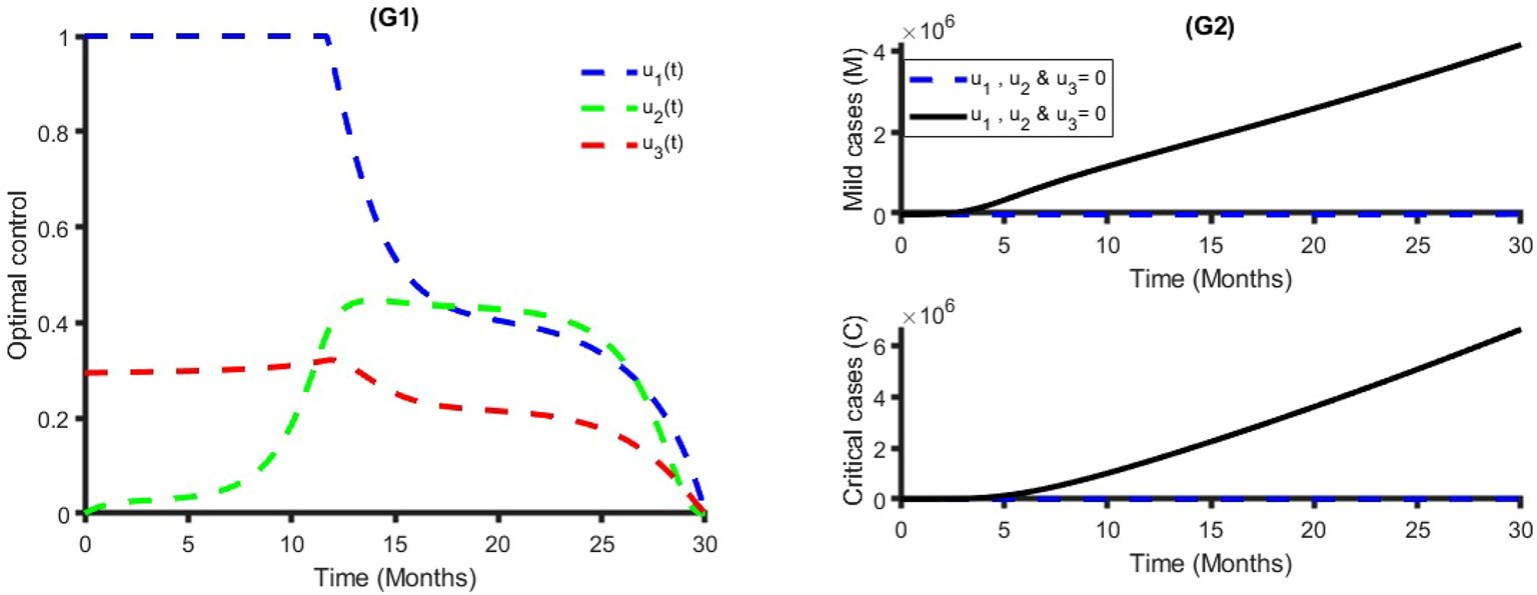
Triple intervention strategy and its impacts on the COVID-19 cases in Bangladesh.

Figure 6 shows the implication of combining the three optimal controls in bringing down the total number of infectious humans to zero in Bangladesh. It is observed that optimal solution has achieved when distancing control strategy $({{\text{u}}}_{1})$ is strictly followed to at the maximum level of 100% for around 10 months, while the vaccination and treatment control strategies $({{\text{u}}}_{2}, {{\text{u}}}_{3})$ are at a maximum level above 40% and 30%, respectively. It can be seen that the combination of the three control strategies is significantly more effective in decreasing the spread of COVID-19 compared to implementing each control strategy individually, which is consistent with the previous modeling studies^32,64,65^.

### Cost-effective analysis

It is essential to identify the most cost-effective scheme for transmission, vaccination, and treatment control as well as their combination control policies to optimally mitigate the spread of COVID-19 at the possible minimum cost. This is achieved by correlating the differences among each intervention’s costs and consequences; acquired by assessing the incremental cost-effective ratio (ICER), which is demarcated as the extra cost per further intervention effect. Incrementally, when analysing two or more competing intervention strategies, one intervention is related to the next less operative option. The total difference in intervention costs gives the ICER numerator, active COVID-19 cases averted costs and averted output losses if applicable, between each setting and starting point. The ICER denominator is the difference in the total number of active COVID-19 cases averted. Hence, the following formula acquires the ICER:

13 $${\text{ICER}}=\frac{\text{Difference in total cost between control strategies}}{\text{Difference in total number of active cases averted by control strategies}}$$

We also completed the average cost-effectiveness ratio (ACER), which assesses the effectiveness of a particular intervention's performance. The ACER is the ratio between the total cost incurred and the total number of active COVID-19 cases averted by that policy. This is calculated by

14 $${\text{ACER}}=\frac{\text{Total cost}}{\text{Total active cases averted}}$$

The total cost for each transmission, vaccination, and treatment implementation and mutual effort of the optimal control strategy is obtainable from the objective function (7). The cases are averted by computing the difference between infectious individuals with and without control strategy.

Let, ${{\text{S}}}_{1}, {{\text{S}}}_{2}$ and ${{\text{S}}}_{3}$ respectively represent a single transmission control strategy ${{\text{u}}}_{1}({\text{t}})$, single vaccination control strategy ${{\text{u}}}_{2}({\text{t}})$ and single-treatment control strategy ${{\text{u}}}_{3}({\text{t}})$. Table 2 summaries the ICER and ACER for each control variable ${{\text{u}}}_{1}\left({\text{t}}\right), {{\text{u}}}_{2}({\text{t}})$ and ${{\text{u}}}_{3}({\text{t}})$ in increasing order of the total infection averted. The ICER and ACER results for ${{\text{S}}}_{1}, {{\text{S}}}_{2}$ and ${{\text{S}}}_{3}$ are calculated using (13) and (14) shown in Table 2.

**Figure 7:**
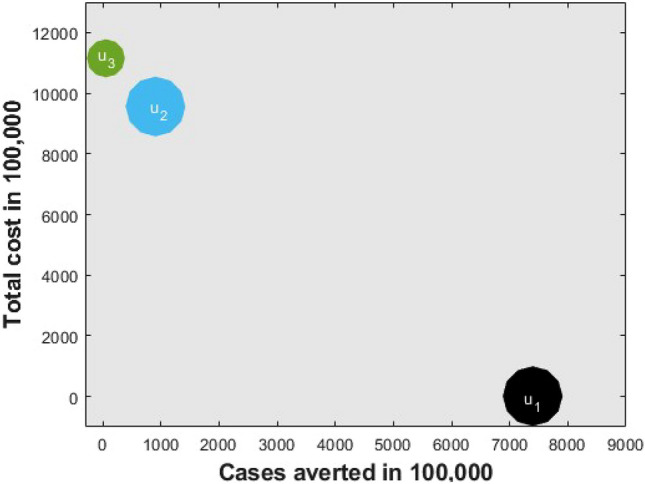
Comparing cost-effective analysis among best single intervention strategies.

Comparing ${{\text{S}}}_{1}, {{\text{S}}}_{2}$ and ${{\text{S}}}_{3}$ in Table 2 and Fig. 7, it is seen that transmission $({{\text{S}}}_{1})$ control strategy is the most cost-effective, which reduces a significant number of COVID-19 cases with a low cost compared to vaccination $({{\text{S}}}_{2})$ and treatment $({{\text{S}}}_{3})$ individually, while ${{\text{S}}}_{3}$ is the least cost-effective intervention strategy among them.

**Figure 8:**
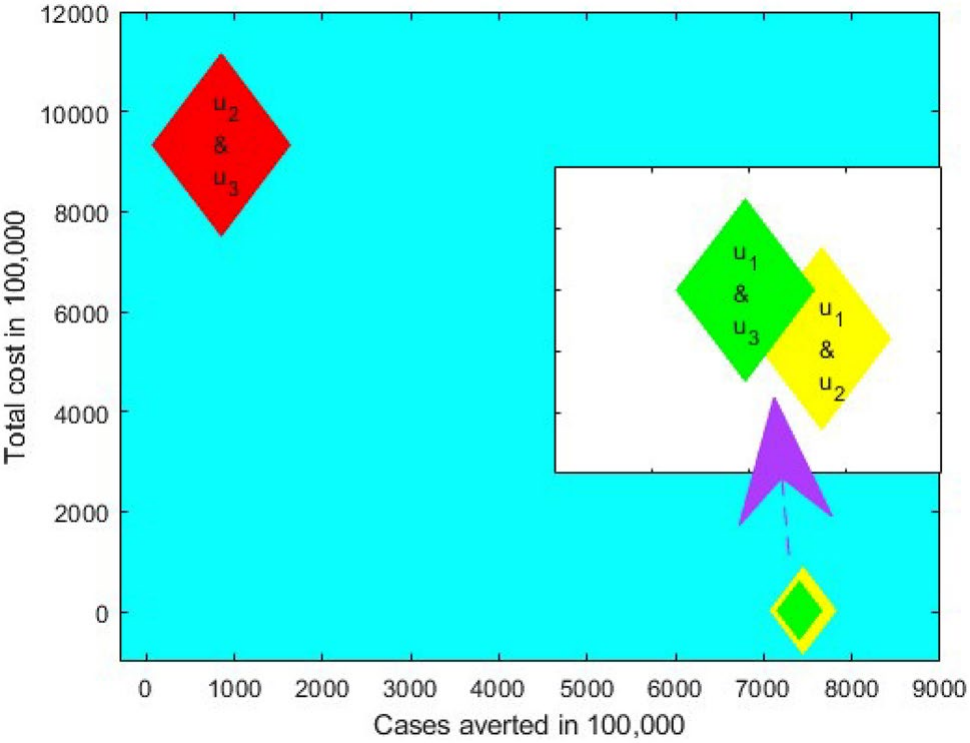
Comparing cost-effective analysis among best double intervention strategies.

Table 3 and Fig. 8 represent the double interventions strategies which include a combination of transmission control and vaccination $({{\text{S}}}_{12})$, transmission control and treatment $({{\text{S}}}_{13})$ as well as vaccination and treatment $({{\text{S}}}_{23})$. Each of the interventions resulted in decreasing the number of COVID-19 cases and relative cost. The analysis shows that a combination of transmission control and vaccination $({{\text{S}}}_{12})$, is the best dual intervention strategy for reducing the number of COVID-19 cases and the minimum cost in Bangladesh (see Table 3 and Fig. 8). Alternative, the combination of transmission control and treatment $({{\text{S}}}_{13})$ is another option.

**Table 3:** ICER and ACER in the order of COVID-19 cases averted by double control measures.

Control measures	Total infected averted	Total cost	ICER	ACER
${{\text{S}}}_{12}$	$7.4377\times {10}^{8}$	$1.2861\times {10}^{6}$	$1.73\times {10}^{-3}$	$1.73\times {10}^{-3}$
${{\text{S}}}_{13}$	$7.3976\times {10}^{8}$	$1.2900\times {10}^{6}$	$-9.73\times {10}^{4}$	$1.74\times {10}^{-3}$
${{\text{S}}}_{23}$	$8.5338\times {10}^{7}$	$9.3250\times {10}^{8}$	$-1.42$	$1.09\times {10}^{-1}$

**Figure 9:**
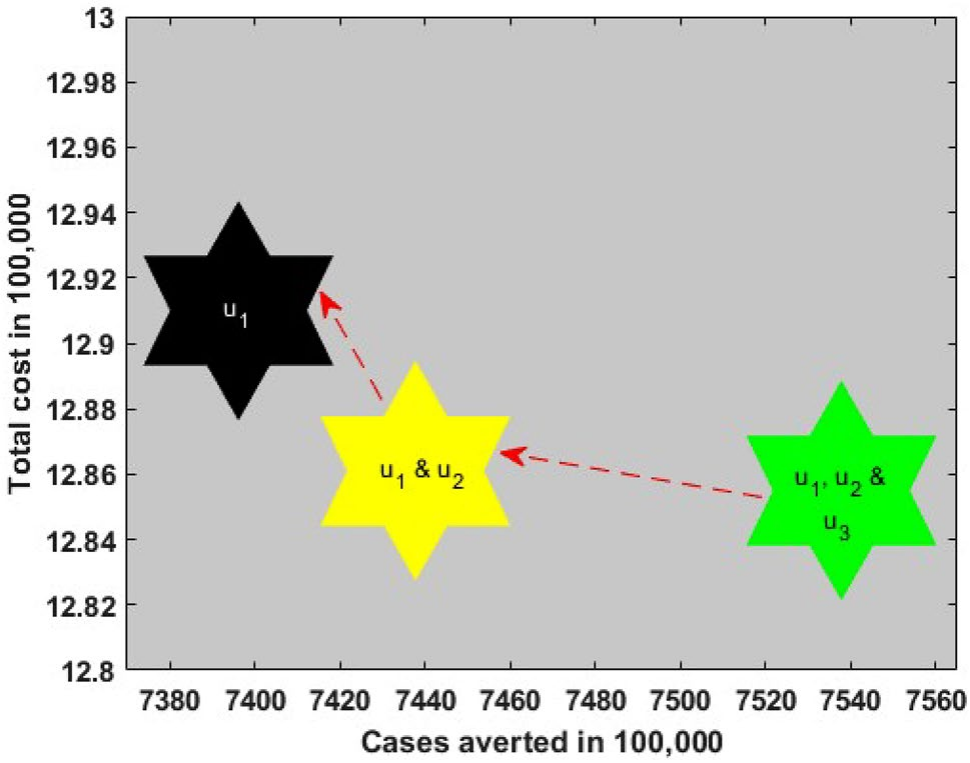
Comparing cost-effective analysis among best single, double, and triple intervention strategies.

Finally, ${{\text{S}}}_{123}$ represents the triple intervention strategy which includes a combination of transmission control ${{\text{u}}}_{1}({\text{t}})$, vaccination ${{\text{u}}}_{2}({\text{t}})$ and treatment ${{\text{u}}}_{3}({\text{t}})$. Under this strategy, the total number of COVID-19 cases reduces enormously with minimum cost over 30 months period due to the combination of triple interventions. We also compared all the scenarios with each other to know which is the most effective (see Table 4 and Fig. 9). Our finding suggests that the combination of triple interventions is the most cost-effective within the best control strategy, which reduces the massive number of COVID-19 cases in Bangladesh. However, another scenario in Table 4 and Fig. 9 can be considered depending on the availability of funds.

**Table 4:** Selecting the best control strategy.

Best control strategy	Total infected averted	Total cost	ICER	ACER
${{\text{u}}}_{1}, {{\text{u}}}_{2}\mathrm{ and }{{\text{u}}}_{3}({{\text{S}}}_{123})$	$7.5378\times {10}^{8}$	$1.2855\times {10}^{6}$	$1.71\times {10}^{-3}$	$1.71\times {10}^{-3}$
${{{\text{u}}}_{1}\mathrm{ and }{{\text{u}}}_{2} ({\text{S}}}_{12})$	$7.4377\times {10}^{8}$	$1.2861\times {10}^{6}$	$-5.99\times {10}^{-5}$	$1.73\times {10}^{-3}$
${{\text{u}}}_{1} ({{\text{S}}}_{1})$	$7.3961\times {10}^{8}$	$1.2910\times {10}^{6}$	$-1.18\times {10}^{-3}$	$1.75\times {10}^{-3}$

## Discussion

Currently, COVID-19 is one of the most persistent public health problems in Bangladesh. Overall, the transmission dynamics and epidemiology of COVID-19 in Bangladesh are poorly understood. The government of Bangladesh started numerous intervention programs, including non-pharmaceutical and pharmaceutical, to eliminate COVID-19. Although COVID-19 control in Bangladesh has expressively proceeded—improved treatment and vaccination coverage, transmission control, adequate capacity, and guidelines—more effort is essential. To diminish COVID-19 incidence, prevalence, and deaths in Bangladesh, we need to recognize the risk factors for increasing COVID-19 cases.

In this paper, we developed a COVID-19 model in Bangladesh to understand the transmission dynamics of COVID-19 in Bangladesh. We derived the basic reproduction number and assessed the role of reproduction number on the dynamics of COVID-19. We performed mesh and contour plots to explore the impact of different parameters on the basic reproduction number. Our investigation led to the explanation that, of the adaptable parameters, the transmission rate had a positive correlation with the basic reproduction number of COVID-19. This associate’s parameter has the highest impact on COVID-19 dynamics and strongly recommends that investments in public health responses that focus on transmission control should be the foundation of enhanced COVID-19 control. We calibrated our model with COVID-19 incidence data in Bangladesh to estimate some model parameters using least-square methods.

We implemented optimal control investigation via Pontryagin’s Maximal principle^61^ and formulated the optimal control strategies for reducing the COVID-19 epidemic in Bangladesh. Three different control strategies were assumed (single, double, and triple) from the combination of transmission control, treatment, and vaccination and were examined to measure their cost-effectiveness.

Between the three single-control interventions, the transmission control strategy is the most cost-effective for reducing the number of COVID-19 cases in Bangladesh. The vaccination control strategy seems to be more effective than the treatment control strategy. Therefore, when only one control strategy is considered, our outcomes recommend that the Ministry of Health in Bangladesh should increase transmission control interventions, dropping transmission between infectious and susceptible individuals.

Combinations with transmission control achieved best within the three-dual-control strategies, and incorporating vaccination control is the most cost-effective and more quickly decreases COVID-19 cases compared to other double intervention strategies. In view of the struggle of employing transmission measures which includes a high social cost and high population density in Bangladesh, pharmaceutical control which contains vaccination and treatment control strategies, should be measured. Therefore, if double control strategies are considered, we suggest that transmission control should be involved. If transmission control is employed effectively, the Ministry of Health in Bangladesh can succeed in the elimination goal with fewer pharmaceutical control practices. Mutual vaccination and treatment control strategy is also advisable if the transmission is infeasible. From the investigation of all the control policies, we found that the most cost-effective control is the triple control strategy, followed by the dual control strategy and single control.

Optimal control approaches have been implemented in other endemic locations to reduce the number of COVID-19 cases and the intervention operation costs^66–68^. Earlier studies show that the transmission control approach is the best approach for the single intervention policy to decline the number of COVID-19 cases and intervention budgets^21,69^, which is similar to our results. In our study, we incorporate the influence of a double-dose vaccination strategy to conduct a cost-effective analysis, unveiling significant implications that accurately reflect the real dynamics of the COVID-19, but other study only considered single dose vaccination strategy^66^. We also considered two important features including direct link between vaccination and latently population, and practical healthcare cost by separation of infections into Mild and Critical cases. In addition, our study displays that transmission control and vaccination are the best preference for the three double-control policies, which is similar to^54^. Our main outcome in this study is that the triple control policy, which contains transmission control, vaccination, and treatment together, is the most impactful and cost-effective strategy for reducing the number of COVID-19 cases. Our result also recommends that effort on a single control strategy will not intensely affect the drop in COVID-19 cases in Bangladesh, whereas combining two or more control strategies concurrently will decline the burden of COVID-19 in Bangladesh, which is found to be consistent with earlier modelling studies^57,69^.

In Bangladesh setting, the current COVID-19 surveillance system may not capture every case, introducing potential bias into our estimates due to underreporting. To mitigate this, it is imperative to implement more refined data collection methods, enhancing the accuracy of COVID-19-related information. The acquisition of precise data not only improves estimation quality but also strengthens the foundation for informed decision-making. Consequently, decisionmakers should be mindful of the potential underreporting bias as they scrutinize our findings for comprehensive and reliable insights.

## Conclusion

In summary, our study sheds light on the critical challenges posed by COVID-19, a global infectious disease responsible for a significant global health burden. Despite universal control measures implemented by governments worldwide, the need for more precise and cost-effective interventions remains evident. Utilizing a mathematical model fitted to Bangladesh's COVID-19 data, we delved into the transmission dynamics, deriving reproduction numbers and employing mesh and contour plots to assess parameter impacts. Within an optimal control framework, our evaluation of single and combination intervention strategies—transmission control, treatment, and vaccination—revealed enhanced transmission control as the most cost-effective approach for rapidly reducing COVID-19 cases in Bangladesh. Leveraging Pontryagin's Maximal Principle, we formulated optimal control strategies to curtail the epidemic. Our findings advocate for a three-pronged intervention strategy integrating transmission control, treatment, and vaccination, proving more cost-effective than single or double interventions. Notably, the vaccination strategy demonstrated greater efficacy than treatment alone. The implementation of these findings underscores the importance of resource availability and policymaker decisions in the ongoing battle against COVID-19.

The forthcoming research will centre on the significance of enhancing household and public awareness as a critical element in controlling the transmission of COVID-19. Consequently, there is a need for additional investigations to ascertain more pertinent and precise policy strategies aimed at eradicating COVID-19 from communities. Accordingly, we suggest that future endeavours should concentrate on determining the optimal policy measures at the local level to manage the transmission of COVID-19 effectively. Therefore, our future research will centre on (i) Investigating the cost-effective analysis for reducing human-to-human transmission of COVID-19 and compare different strategies both local and national levels to control the COVID-19 transmission (iii) Exploring the cost-effectiveness of intervention policies in local and national levels considering differences in healthcare infrastructure, population density, and socio-economic factors (Supplementary Information S1).

### Supplementary Information

Supplementary Information.

## Data Availability

Data will be made available on reasonable request. All data were compiled from the publicly available website https://dashboard.dghs.gov.bd/pages/covid19.php.
