# Supplementary material for: Cost-effectiveness analysis of COVID-19 intervention policies using a mathematical model: an optimal control approach
Source: Sci Rep. 2024 Jan 4;14:494. doi: 10.1038/s41598-023-50799-6 (PMC10766655; doi:10.1038/s41598-023-50799-6)
Supplement: Supplementary file 1 — Supplementary Information. [file 41598_2023_50799_MOESM1_ESM.docx]

Supplementary materials

Cost-effectiveness analysis of COVID-19 intervention policies using a mathematical model: An optimal control approach

Md Abdul Kuddus^1^, Anip Kumar Paul^1^ and Thitiya Theparod^2*^

^1^Department of Mathematics, University of Rajshahi, Rajshahi-6205, Bangladesh

^2^Department of Mathematics, Mahasarakham University, Maha Sarakham 44150, Thailand

*Corresponding author: Thitiya Theparod

e-mail: thitiya.t@msu.ac.th

%Model_runner

clc

clear all

global xdata ydata

%Bangladesh data

time_step = 0.0001;

cases= [51;7616;39492;89566;109908;90249;63976;56560;58579;58472;30058;17018;29858;91604;37998;112718];

cumcases=cumsum(cases)

months=[0;1;2;3;4;5;6;7;8;9;10;11;12;13;14;15];

%Data to fit to::

xdata=months; ydata=cumcases;

size(xdata)

size(ydata)

%plot(pre,years)

%%%%Fitting

options=optimoptions('fmincon','Algorithm','interior-point','DerivativeCheck'...

,'on','TolX',1e-12,'TolFun',1e-12,'MaxIter',10000,'MaxFunEvals',10000);

%Initial guess

guess=[0.00008, 0.1,0.1,0.1,0.001,0.002];

lb=[0,0,0,0,0,0];%Lower bound

ub=[1,1,1,2,1,1]; %Upper bound

[result,lik1,exit1]=fmincon(@myObjective,guess,[],[],[],[],lb,ub,[],options);

exit1;

result

%get output

myoutput1=SolveTB(result,xdata)

figure(3)

plot(xdata,myoutput1,'g','linewidth',5)

%plotshaded(xdata',[myoutput1'+myoutput1'.*0.2; myoutput1'-myoutput1'.*0.2],'b')

hold on

plot(xdata,ydata,'bO','linewidth',5)

%%

month = {'Mar','May','Jul', 'Sep', 'Nov', 'Jan', 'Mar', 'May','Jul'};

set(gca, 'xticklabel',month)

set(gca,'YTickLabel',[])

set(gca,'YTick',[])

xlabel('Month (2021-2022)')

ylabel('Cumulative cases')

xlim([0 16])

ylim([0 11*10^5])

%axis equal

%%%Define the objective function

function yerr=myObjective(guess)

simu=SolveTB(guess,xdata);

size(simu)

size(ydata)

yerr=sum((simu-ydata).^2);

end

function myoutput=SolveTB(guess,xvalues)

beta=guess(1);

mu=1/70;

omega1=guess(2);

omega2=guess(3);

gamma1=0.02;

gamma2=0.01;

phi=0.3;

rho=0.2;

delta=0.125;

eta=guess(4);

sigma=0.9;

kappa=0.80;

alpha1=guess(5);

alpha2=guess(6);

%lamdha=159000000;

N=164689383;

R0=(N*beta*guess(2))./(mu).*(gamma1+mu)*mu

runs = length(omega1);

stop_time = 16;

time_step = 0.001;

S_all = zeros(stop_time / time_step + 1, runs);

V1_all = zeros(stop_time / time_step + 1, runs);

V2_all = zeros(stop_time / time_step + 1, runs);

L_all = zeros(stop_time / time_step + 1, runs);

M_all = zeros(stop_time / time_step + 1, runs);

C_all = zeros(stop_time / time_step + 1, runs);

R_all = zeros(stop_time / time_step + 1, runs);

for it = 1 : runs, it

%alpha = alpha_values(it);

param = zeros(16,1);

param(1) = beta;

param(2) = mu;

param(3) = R0;

param(4) = omega1;

param(5) = omega2;

param(6) = gamma1;

param(7) = gamma2;

param(8) = phi;

param(9) = N;

param(10) = rho;

param(11) = delta;

param(12) = eta;

param(13) = sigma;

param(14) = kappa;

param(15) = alpha1;

param(16) = alpha2;

V10=200;

V20=100;

L0 =2000;

M0 =(guess(2)*L0)/(phi+gamma1+mu);

C0=(guess(3)*L0+phi*M0)/(gamma2+delta+mu);

R0=0000;

S0 = N -V10-V20- L0 - M0 - C0 - R0;

initial_x = [S0, V10, V20, L0, M0, C0, R0];

% vectors to store simulation results

time = [];

S = [];

V1 = [];

V2 = [];

L = [];

M = [];

C = [];

R = [];

options = odeset('NonNegative',1:7);

% integrate the ODE system for the first untreated period

[t, y] = ode45(@(t, x) model_equations(t, x, param), ...

xvalues, ...

initial_x, options);

t = real(t);

y = real(y);

% iterative sotrage of model outputs

S = y(:, 1);

V1 = y(:, 2);

V2 = y(:, 3);

L = y(:, 4);

M = y(:, 5);

C = y(:, 6);

R = y(:, 7);

cumucases=(guess(2)+guess(3))*L;

myoutput=(cumucases)

end

end

% Runs model function

function der = model_equations(t, x, parameter)

beta=parameter(1);

mu=parameter(2);

R0=parameter(3);

omega1=parameter(4);

omega2=parameter(5);

gamma1=parameter(6);

gamma2=parameter(7);

phi=parameter(8);

N=parameter(9);

rho=parameter(10);

delta=parameter(11);

eta=parameter(12);

sigma=parameter(13);

kappa=parameter(14);

alpha1=parameter(15);

alpha2=parameter(16);

S = x(1);

V1 = x(2);

V2 = x(3);

L = x(4);

M = x(5);

C = x(6);

R = x(7);

dS=mu*N+rho*V1+delta*C-(beta*(M+C)*S)-eta*S-mu*S;

dV1=eta*S-(rho+sigma+alpha1+mu)*V1;

dV2=sigma*V1-(kappa+alpha2+mu)*V2;

dL=(beta*(M+C)*S)-(omega1+omega2+mu)L+(alpha1+alpha2)*L;

dM=omega1*L-(phi+gamma1+mu)*M;

dC=omega2*L+phi*M-(gamma2+delta+mu)*C;

dR=gamma1*M+omega2*C+kappa*V2-mu*R;

der = [dS; dV1; dV2; dL; dM; dC; dR];

end
